# NK cell transfer overcomes resistance to PD-(L)1 therapy in aged mice

**DOI:** 10.1186/s40164-024-00511-9

**Published:** 2024-05-09

**Authors:** Junlei Hou, Shuanglong Xie, Jianbao Gao, Tao Jiang, Enjian Zhu, Xuezhi Yang, Zheng Jin, Haixia Long, Anmei Zhang, Fei Yang, Lujing Wang, Haoran Zha, Qingzhu Jia, Bo Zhu, Xinxin Wang

**Affiliations:** 1 Institute of Cancer, Xinqiao Hospital, Third Military Medical University, Chongqing, 400037 China; 2 Shanghai Pulmonary Hospital, Shanghai, 200082 China; 3 Department of Oncology, PLA Rocket Force Characteristic Medical Center, Beijing, 100088 China; 4 Jinfeng Laboratory, Chongqing, 401329 China

**Keywords:** Aging, Cancer immunotherapy, Natural killer cells, Dendritic cells

## Abstract

**Background:**

Cancer is the leading cause of death among older adults. Although the integration of immunotherapy has revolutionized the therapeutic landscape of cancer, the complex interactions between age and immunotherapy efficacy remain incompletely defined. Here, we aimed to elucidate the relationship between aging and immunotherapy resistance.

**Methods:**

Flow cytometry was performed to evaluate the infiltration of immune cells in the tumor microenvironment (TME). In vivo T cell proliferation, cytotoxicity and migration assays were performed to evaluate the antitumor capacity of tumor antigen-specific CD8^+^ T cells in mice. Real-time quantitative PCR (qPCR) was used to investigate the expression of IFN-γ-associated gene and natural killer (NK)-associated chemokine. Adoptive NK cell transfer was adopted to evaluate the effects of NK cells from young mice in overcoming the immunotherapy resistance of aged mice.

**Results:**

We found that elderly patients with advanced non-small cell lung cancer (aNSCLC) aged ≥ 75 years exhibited poorer progression-free survival (PFS), overall survival (OS) and a lower clinical response rate after immunotherapy. Mechanistically, we showed that the infiltration of NK cells was significantly reduced in aged mice compared to younger mice. Furthermore, the aged NK cells could also suppress the activation of tumor antigen-specific CD8^+^ T cells by inhibiting the recruitment and activation of CD103^+^ dendritic cells (DCs). Adoptive transfer of NK cells from young mice to aged mice promoted TME remodeling, and reversed immunotherapy resistance.

**Conclusion:**

Our findings revealed the decreased sensitivity of elderly patients to immunotherapy, as well as in aged mice. This may be attributed to the reduction of NK cells in aged mice, which inhibits CD103^+^ DCs recruitment and its CD86 expression and ultimately leads to immunotherapy resistance.

**Supplementary Information:**

The online version contains supplementary material available at 10.1186/s40164-024-00511-9.

## Introduction

Immune checkpoint blockade (ICB), including antibodies targeting PD-(L)1, have revolutionized the landscape of cancer treatment. However, challenges such as an efficacy ceiling of approximately 20% have hindered the full realization of the clinical potential of ICB [1]. Understanding intra-tumoral cell subpopulations and their functions in the context of immunotherapy is crucial for improving immunotherapeutic responses [2–4]. Aging, which plays an important role in cancer pathogenesis, causes perturbations in hematopoietic stem cell differentiation [5, 6] and thymic atrophy [7], inducing profound alterations in innate and adaptive immune cell subsets [8]. However, the influence of aging on the composition and function of immune cells in the tumor microenvironment (TME) and on the efficacy of immunotherapy remains unclear. In clinical trials, elderly patients with cancer are underrepresented, and few studies have been conducted on the effectiveness of ICIs in elderly patients [8, 9]. For example, less than 10% of patients enrolled in a clinical trial of breast cancer were aged ≥ 75 years [9]. Furthermore, the general practice of using young mice (aged 6–8 weeks) in preclinical investigations diverges significantly from the clinical age spectrum of patients. Consequently, conducting a comprehensive investigation into the efficacy of immunotherapy in elderly patients with cancer and the underlying mechanisms of immunotherapy resistance is of paramount importance.

Natural killer (NK) cells are an essential component of the innate immune system and hold central significance within the TME. NK cells exert direct cytotoxic effects on tumor cells by releasing granzyme B and perforin and expressing the Fas ligand (FasL/CD95L) [10, 11]. Consequently, NK cells are extensively utilized in adoptive cell therapy for tumor patients, exhibiting notable efficacy and safety [12, 13]. Moreover, NK cells fulfill an immunomodulatory role by producing cytokines and modulating dendritic cells (DCs), thus promoting adaptive immune responses. Due to the secretion of chemokines such as CCL5, XCL1/2, and FLT3LG, NK cells facilitate DCs recruitment and enhance the efficacy of anti-PD-(L)1 immunotherapy [14, 15]. Despite the therapeutic significance, the specific impact of age-related changes in NK cells on the effectiveness of immunotherapy remains obscure.

Therefore, the objective of this study was to investigate the specific age-related changes in NK cells, and their impact on the response to immunotherapy across various murine tumor models. The results may provide a novel therapeutic regimen by facilitating the development of age-related immunotherapeutic strategies.

## METHODS

### Patient clinical information

This study enrolled 205 patients diagnosed with advanced non-small cell lung cancer (aNSCLC) at Shanghai Lung Hospital from 2015 to 2018. All patients underwent PD-1 blockade therapy. The cohort comprised 191 patients aged < 75 years (younger) and 14 patients aged ≥ 75 years (older). Detailed clinical information is provided in Table S1.

### Mice

Two–three months (young) or 13 months (aged) female C57BL/6 and BALB/c mice were procured from the Chinese Academy of Medical Sciences in Beijing, China. In vivo studies involved random group assignment of mice before experimentation, which daily examinations conducted after that. Sample sizes were determined based on previous experience. All animal experiments were performed in a non-blinded manner, and mice were kept under specific pathogen-free conditions. Housing and treatment protocols adhered to the guidelines established by the Animal Care and Use Committee of the Third Military Medical University (TMMU) in Chongqing, China. The Institutional Animal Care and Use Committee of TMMU approved all animal experiments.

### Cell lines and cell culture conditions

LLC, CT26, and 4T1 cells were obtained from the American Type Culture Collection (ATCC). MC38 cells were provided by Liufu Deng (Shanghai Jiao Tong University, China), while MC38-OVA cells were supplied by Bo Guo (Jinan University, China). These cells were cultured in Dulbecco’s modified Eagle’s medium (DMEM; Gibco, Australia), supplemented with 10% heat-inactivated fetal bovine serum (FBS; Gibco, Australia), penicillin (100 U/mL), and streptomycin (100 U/mL). in an incubator at 37 °C with 5% CO_2_.

### Tumor challenge and treatment

Tumor cells (1 × 10^6^ LLC, 5 × 10^5^ CT26, 5 × 10^5^ 4T1, 5 × 10^5^ MC38, or 5 × 10^5^ MC38-OVA) were suspended in 100 µL of phosphate-buffered saline (PBS). Then, the tumor cells were inoculated subcutaneously into the right flanks of the mice on day 0. Anti-CD8a antibody (200 µg; clone YTS169.4, Bio X Cell) or control IgG2b (200 µg; clone LTF-2, Bio X Cell) was injected intraperitoneally to deplete CD8^+^ T cells on days 0, 3, 6, 9, and 12 post-tumor challenge. Anti-PD-L1 (200 µg; clone 10 F.9G2™, Bio X Cell) or IgG2b control (200 µg; clone LTF-2, Bio X Cell) were injected intraperitoneally on days 4, 7, 10, and 13 post-tumor challenge. Tumor growth was monitored every other day, and the tumor volumes were calculated using the formula: V = (length × width^2^)/2. Mice were sacrificed using cervical spinal cord dislocation at specified time points post-tumor inoculation, and tumor tissues, tumor-draining lymph nodes (TDLNs), and non-draining lymph nodes (NDLNs) were dissociated into single cells for flow cytometry analysis. All experimental procedures were adhered to the Institutional Animal Care and Use Committee of TMMU guidelines.

### Preparation of single-cell suspensions

Tumors and lymph nodes were dissected and then digested with digestion solution, which contained 1 mg/mL collagenase I (Sigma-Aldrich, Germany), 10% FBS (Tianjin TBD Standard, China), and 50 µg /mL DNase I (Invitrogen, USA) in RPMI 1640. Digestion was performed on a shaker at 37 ° C, 148 rpm for 30 min. The spleens were mechanically disrupted and filtered through a 70-µm nylon cell strainer (BD Biosciences, USA).

### Flow cytometry and antibodies

The cells were labeled with Fixable Viability Dye eFluor 780 (eBioscience) to assess viability. To minimize non-specific staining, each 10^6^ cells were co-stained with 0.25 µg TruStain FcX™ (anti-mouse CD16/32) Antibody (clone 93, BioLegend) and anti-mouse antibodies per 100ul for 30 min on ice. These anti-mouse antibodies included anti-CD45 (clone 30-F11, BioLegend), anti-CD3 (clone 17A2, BioLegend), anti-CD8a (clone 53-6.7, BioLegend), anti-CD4 (clone RM4-5, BioLegend), anti-FOXP3 (clone MF-14, BioLegend), anti-CD19 (clone 6D5, BioLegend), anti-CD11b (clone M1/70, BioLegend), anti-Ly6G/Ly6C (Gr1) (clone RB6-8C5, BioLegend), anti-F4/80 (clone BM8, BioLegend), anti-NK1.1 (clone PK136, BioLegend), anti-CD335 (clone 29A1.4, BioLegend), anti-CD49a (clone HMα1, BioLegend), anti-NKG2A (clone 16A11, BioLegend), anti-LAG3 (clone C9B7W, BioLegend), anti-I-A/I-E (clone M5/114.15.2, BioLegend), anti-CD11c (clone N418, BioLegend), anti-CD27 (clone LG.3A10, BioLegend), anti-CD86 (clone GL-1, BioLegend), anti-CD44 (clone NIM-R8, BioLegend), anti-H-2K^b^ (clone AF6-88.5, BioLegend), anti-CD103 (clone 2E7, BioLegend), anti-CD107a (clone 1D4B, BioLegend), anti-Ki67 (clone 11F6, BioLegend), anti-IFN-γ (clone XMG1.2, BioLegend), and anti-TNF-α (clone MP6-XT22, BioLegend) antibodies. Phycoerythrin-labeled MHC class I (H-2K^d^) tetramer carrying the ovalbumin peptide SIINFEKL was used to evaluate OVA-specific CD8^+^ tumor-infiltrating lymphocytes. Unless otherwise indicated, surface staining was conducted on ice in PBS containing 2% FBS for 30 min. Ki-67 staining was performed with a Foxp3/Transcription Factor Staining Buffer Set (cat#:00-5523-00, eBioscience). Following the manufacturer’s instructions, intracellular cytokine staining involved cell stimulation with phorbol 12-myristate 13-acetate (50 ng/mL, Sigma-Aldrich) plus ionomycin (500 ng/mL, Abcam) alongside GolgiPlug Protein Transport Inhibitor containing Brefeldin A (BD Biosciences). Additionally, cells were stimulated with H-2K^b^-restricted MuLV p15E KSPWFTTL peptide (KSP) (2 µg/mL) (MBL) plus Brefeldin A (1 × solution/mL) (Invitrogen). After 5 h, cells were stained with surface markers, fixed, and permeabilized using Fixation/Permeabilization Solution (BD Biosciences) according to the manufacturer’s instructions. For in vitro NK cell activation, NK cells were stimulated with RMPI 1640 containing IL-12/IL-15 (10 ng/ml) and IL-18 (50 ng/ml) and incubated at 37℃ with 5% CO_2_ for 24 h. BD GolgiStop™ Protein Transport Inhibitor containing Monensin (BD Biosciences) and BD GolgiPlug™ Protein Transport Inhibitor Containing Brefeldin A (BD Biosciences) were added 4 h before the detection. Subsequently, cells were stained with the intracellular cytokine antibodies. Flow cytometry data were acquired using a Gallios flow cytometer (Beckman Coulter) and analyzed by FlowJo software v10 (BD Biosciences). The fluorescence-activated cell sorting (FACS) gating strategy for tumor-infiltrating immune cells is depicted in Fig. S1.

### *In vivo* cytotoxicity assays

Splenocytes were isolated from tumor-free C57BL/6 female mice aged 6–8 weeks. The spleens were mechanically dissociated and filtered through a 70-µm nylon cell strainer (BD Biosciences, USA), followed by erythrocyte lysis using Erythrocyte Lysate (Biosharp, China) to obtain a single-cell suspension. The isolated splenocytes were then divided into aliquots and exposed to either OVA_257 − 264_ peptide (5 µg/mL) or KSPWFTTL irrelevant peptide (5 µg/mL) for 1 h at 37 °C with 5% CO_2_. After incubation, the cells were washed with PBS and stained with CellTrace Violet (CTV) (Invitrogen) for 6 min at 37 °C. Cells pulsed with the OVA_257 − 264_ peptide were labeled with CTV^hi^ (5 µM) as target cells, while those with irrelevant peptide were labeled with CTV^low^ (0.5 µM) as internal controls. Following washing and counting, these cells were mixed at a 1:1 ratio, and intravenously injected into young and aged C57BL/6 mice bearing MC38-OVA tumors. Spleens were extracted 48 h later, and the CTV^lo^/CTV^hi^ cell ratio was assessed using flow cytometry (Beckman Coulter).

### Adoptive NK cell transfer

Briefly, splenocytes were obtained from tumor-free C57BL/6 female mice aged 6–8 weeks or 13 months. Then, NK cells were isolated from splenocytes utilizing the MojoSort™ Mouse NK Cell Isolation Kit (BioLegend) following the manufacturer’s protocols. Subsequently, flow cytometry (BD FACS Arial III) was employed for cell sorting, resulting in a purity exceeding 98% (Fig. S2). On day one post-tumor challenge, congenic recipient mice received intravenous injections of 1 × 10^6^ NK cells.

### T-cell migration assay

Splenocytes were obtained from tumor-free C57BL/6 female CD45.2^+^ OT-I mice aged 6–8 weeks. These cells were then stimulated with the OVA_257 − 264_ (1 µg/mL) peptide in the presence of mouse recombinant IL-2 (30 IU/mL). Following the manufacturer’s protocols, activated OT-I (CD44^+^CD8^+^CD3^+^) cells were subsequently isolated using the EasySep™ Mouse CD8^+^ T Cell Isolation Kit (STEMCELL Technologies) after six days. The isolated cells were labeled with CTV (5 µM). For the in vivo migration assay, 2 × 10^5^ CTV-labeled OT-I cells were intravenously injected into MC38-OVA tumor-bearing mice. Tumors were harvested after 48 h. Data acquisition was performed using a Gallios flow cytometer (Beckman Coulter). The data were analyzed by FlowJo software (version V10).

### *In vivo* T cell proliferation assay

Splenocytes were obtained from tumor-free C57BL/6 female CD45.1^+^ OT-I mice aged 6–8 weeks. Following the manufacturer’s protocols, naïve OT-I (CD44^−^CD62L^+^CD8^+^CD3^+^) cells were isolated using the EasySep™ Mouse Naïve CD8^+^ T Cell Isolation Kit (STEMCELL Technologies) after four or eight days. Subsequently, the cells were labeled with CTV (5 µM). For the T cell proliferation assay, 2 × 10^5^ CTV-labeled OT-I cells were intravenously injected into MC38-OVA tumor-bearing mice. Tumors were harvested after 72 h. Data acquisition was performed using a Gallios flow cytometer (Beckman Coulter). The data were analyzed by FlowJo software (version V10).

### Real-time quantitative PCR

RNA extraction was performed with the RNeasy Mini Kit (Qiagen), and reverse transcription of 1 µg total RNA was carried out using a LightCycler (Roche Diagnostics). The primers utilized for RT-qPCR analysis are detailed in Table S2.

### Survival analysis

Details regarding the immuno-oncology (IO) cohort were previously described in the Patient clinical information section. In this cohort, patients were categorized into two groups, with the age of 75 as the cutoff point. We used GraphPad Prism 7.0 (GraphPad Software) for the Kaplan‒Meier survival analysis.

### Statistical analysis

Each experiment was replicated 2 to 3 times. GraphPad Prism 7.0 (GraphPad Software) was utilized for statistical analyses. Two-tailed Student’s t-tests or Mann-Whitney U tests were employed to assess p-values for the difference between the two groups, as specified. One-way or two-way analysis of variance (ANOVA) was applied to compute p-values for distinctions among multiple groups. Survival curves underwent analysis using the log-rank (Mantel–Cox) test.

## RESULTS

### Age-related disparities in survival and immunotherapeutic response in patients with aNSCLC

**Fig. 1:**
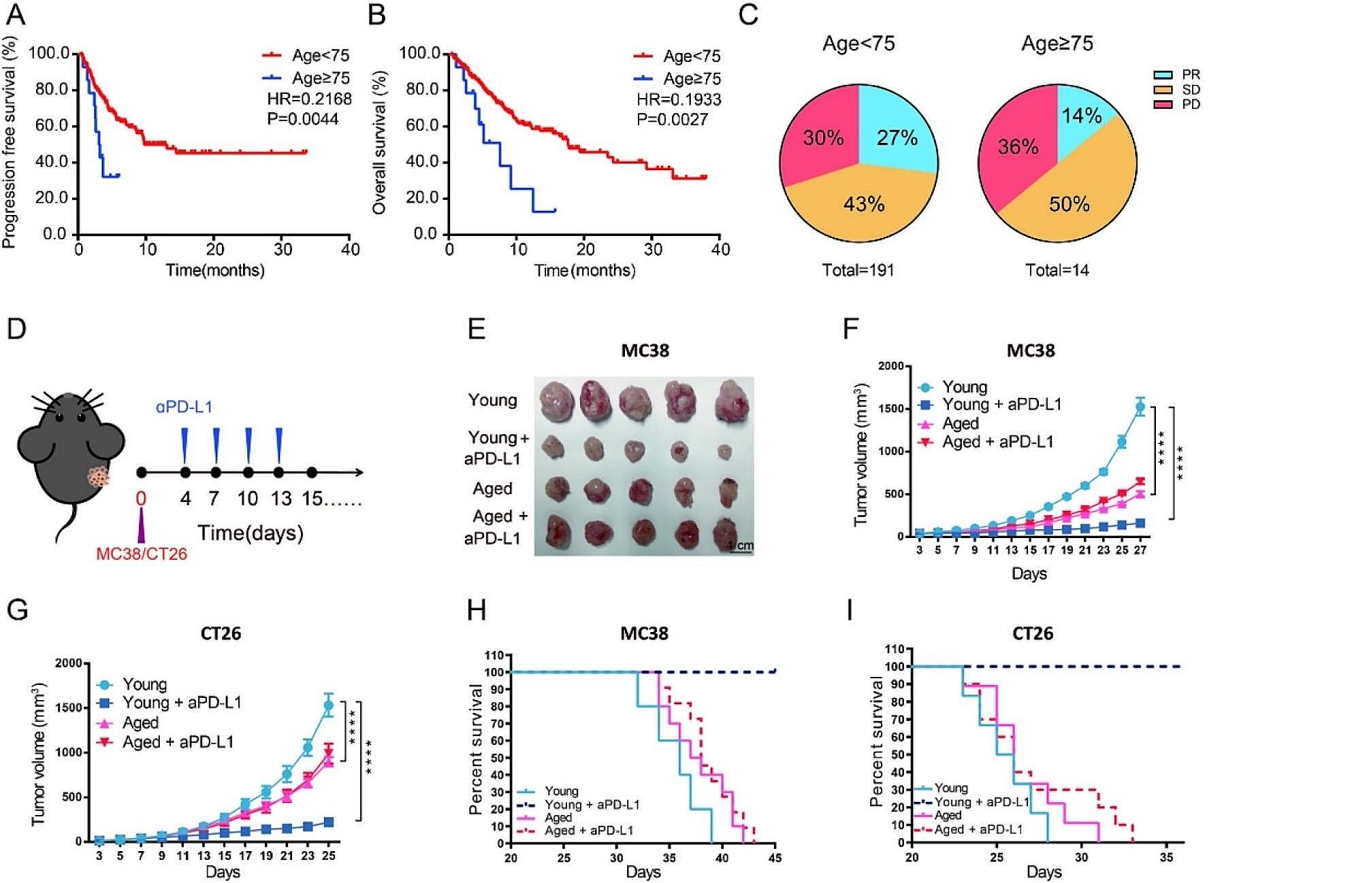
Aging reduces the effectiveness of anti-PD-(L)1 therapy. (A and B) Kaplan-Meier curves illustrating PFS and OS among patients aged < 75 years (*n* = 191) and ≥ 75 years (*n* = 14) with aNSCLC who underwent PD-1 blockade therapy. (C) Pie charts illustrating the disparity in objective response rate by age. PR, partial response, (younger patients: 51/191; older patients: 2/14); SD, stable disease, (younger patients: 83/191; older patients: 7/14); PD, progressive disease, (younger patients: 57/191; older patients: 5/14). (D–I) Analysis of tumor growth in MC38 and CT26 tumor-bearing mice. Young and aged C57BL/6 mice received subcutaneous injection of MC38 cells into their right flanks. Young and aged BALB/c mice were inoculated subcutaneously with CT26 cells into their right flanks. Anti-PD-L1 or control IgG2b was intraperitoneally injected on days 4, 7, 10, and 13 post-tumor challenge. (D) Experimental schedule; and (E) representative tumor images (Day 27 post-tumor challenge). (F) Individual growth curves of MC38 (*n* = 5 mice per group); (G) CT26 (*n* = 7 mice per group), (H) survival curves of MC38 (*n* = 5–10 mice per group) and (I) CT26 (*n* = 5–10 mice per group). Two-way ANOVA (F and G), or Kaplan-Meier analysis followed by log-rank tests (A-B and H-I) were utilized to evaluate statistical significance (^*^p < 0.05, ^**^p < 0.01, ^***^p < 0.001, ^****^p < 0.0001). Error bars represent *SEM*

This study examined the association between age and two critical clinical endpoints, progression-free survival (PFS) and overall survival (OS), in an independent IO cohort of 205 patients with aNSCLC. Notably, patients aged 75 and above (older) exhibited significantly shorter PFS (3.1 months vs. 13.1 months, *p* = 0.0044) and OS (7.6 months vs. 17.6 months, *p* = 0.0027) compared to those under 75 (younger) (Fig. 1A–B). Additionally, the response to immunotherapy varied by age, with a significantly lower partial response (PR) of the older group compared to the younger group (Fig. 1C). In addition, age was an independent risk factor for OS and PFS according to the multivariate Cox regression analysis (Fig. S3).

To determine the replicability of our observed age-associated differences in immunotherapy responses in patients with aNSCLC, we established preclinical models using two immunotherapy (anti-PD-1/PD-L1) sensitive mouse colon cancer cell lines MC38 and CT26. These cells were subcutaneously injected into young (2–3 months old) and aged (> 13 months old) female C57BL/6 or BALB/c mice (Fig. 1D). Moreover, we observed a robust anti-tumor effect of anti-PD-L1 in young mice, but this effect was not observed in aged mice (Fig. 1E–I). These findings highlight the significant resistance of aged mice to anti-PD-L1 therapy compared to their younger counterparts, consistent with clinical observations among patients with aNSCLC.

### Anti-PD-L1 therapy fails to activate an effective anti-tumor immune response in aged mice

**Fig. 2:**
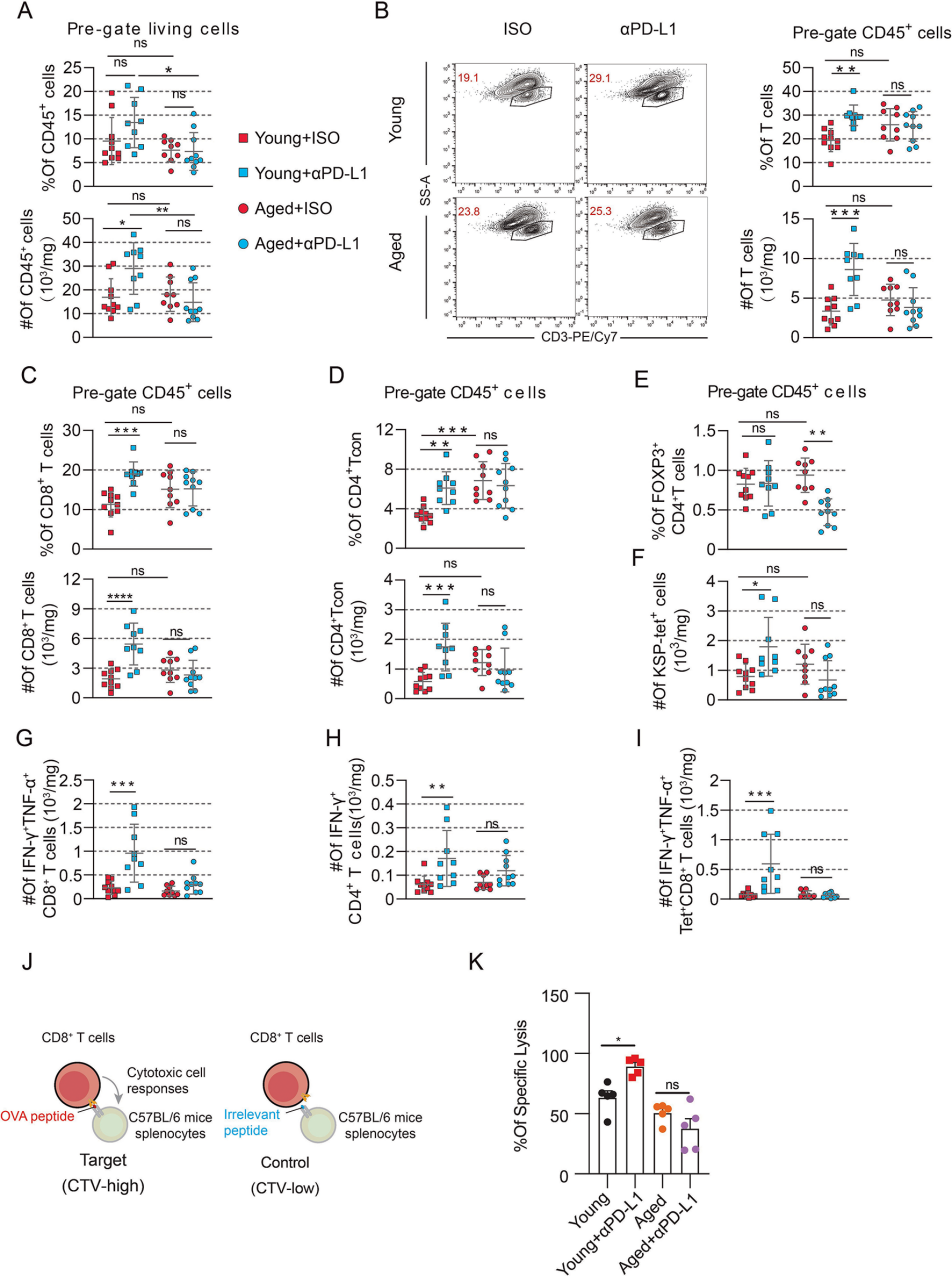
Ineffectiveness of anti-PD-L1 therapy in activating anti-tumor immune response in aged mice. (A-I) For PD-L1 blockade, anti-PD-L1 or control IgG2b were injected intraperitoneally on days 4, 7, 10, and 13 after the tumor challenge. Mice were euthanized on day 15, and then the tumor was dissociated into single cells for flow cytometry analysis. (A) Quantification of the proportion and absolute number of CD45^+^ cells in living cells (*n* = 9–10 mice per group). (B) Representative flow plots and a summary of the proportion and absolute number of CD3^+^ T cells in CD45^+^ cells (*n* = 9–10 mice per group). (C) Quantification of CD3^+^CD8^+^ T cells proportion and absolute number in CD45^+^ cells (*n* = 9–10 mice per group). (D) Quantification of CD3^+^Foxp3^−^CD4^+^ Tcon proportion and absolute number in CD45^+^ cells (*n* = 9–10 mice per group). (E) Quantification of Foxp3^+^CD4^+^ T cells proportion in CD45^+^ T cells (*n* = 9–10 mice per group). (F) Quantification of KSP-tet^+^ CD8^+^ T cells absolute number in CD8^+^ T cells (*n* = 9–10 mice per group). (G) The absolute number of IFN-γ and TNF-α co-expression in CD8^+^ T cells (*n* = 9–10 mice per group). (H) Quantification of IFN-γ absolute number of CD4^+^ T cells (*n* = 9–10 mice per group). (I) The absolute number of IFN-γ and TNF-a co-expression in KSP-specific CD8^+^ T cells (*n* = 9–10 mice per group). (J and K) An in vivo cytotoxicity assay was conducted in young and aged MC38-OVA tumor-bearing mice. A 1:1 mixture of CTV^hi^-labelled OVA-loaded splenocytes and CTV^low^-labelled irrelevant peptide-loaded control cells was co-injected via the tail vein into MC38-OVA-bearing young and aged mice. The ratio of CTV^low^ cells to CTV^hi^ cells in the spleens was analyzed after 48 h. (J) Experimental schedule; and (K) the quantification of percent cell death (*n* = 5 mice per group). In A–I and K, one-way analysis of variance (ANOVA) was used to evaluate statistical significance (*p < 0.05, **p < 0.01, ***p < 0.001, ****p < 0.0001). Error bars represent *SD*

To gain deeper insights into the effectiveness of anti-PD-L1 therapy in activating antitumor immune responses across different age groups, we evaluated immune cell subsets and their functional dynamics within the TME. The absolute number of immune cells (CD45^+^) increased within the TME of young mice following anti-PD-L1 therapy. In contrast, no significant increase was observed in aged mice (Fig. 2A). Notably, both the proportion and absolute number of immune cells were higher in young mice compared to aged mice after anti-PD-L1 therapy (Fig. 2A). Subsequently, we comprehensively examined the infiltration and function of T cell subsets across various age groups using flow cytometry. In young mice, anti-PD-L1 therapy markedly increased both the proportion and quantity of CD3^+^ T cells within the TME. In contrast, this phenomenon was not observed in aged mice (Fig. 2B). Anti-PD-L1 therapy could also increase the infiltration of CD8^+^ T cell and CD4^+^ Tcon (CD3^+^FOXP3^-^CD4^+^) (Fig. 2C–D). However, anti-PD-L1 therapy resulted in a decrease in the infiltration of regulatory T cells (Tregs) in aged mice. Still, it was not observed in young mice (Fig. 2E). Specifically, anti-PD-L1 therapy could amplify tumor antigen-specific CD8^+^ T cells in young mice, but not in aged mice (Fig. 2F), and the ability to secrete killer cytokines IFN-γ and TNF-ɑ of T cells is also significantly improved in young mice (Fig. 2G–I). Additionally, in vivo experiments have confirmed that tumor antigen-specific CD8^+^ T cells in young mice exhibited stronger cytotoxicity than those in aged mice after anti-PD-L1 therapy (Fig. 2J–K). These findings suggest that aged tumor-bearing mice do not benefit from anti-PD-L1 therapy, most likely due to ineffective activation of tumor antigen-specific immunity.

### Decreased number of tumoral NK cells in aged mice

The complex TME comprises tumor cells, immune cells, fibroblasts, endothelial cells, and their secreted cytokines, which may lead to ICB resistance. Due to IFN-γ-associated signaling is the main feature of the effectiveness of anti-PD-(L)1 therapy [16], we firstly assessed the change of IFN-γ-related gene profiles from both young and aged mice. However, there were similar IFN-γ-associated signaling profiles between young and aged mice (Fig. S4).

Secondly, we explored the infiltration of three major immunosuppressive cell types in MC38 tumors: tumor-associated macrophages (TAMs), myeloid-derived suppressor cells (MDSCs) and Tregs. No disparities in their proportions were detected (Fig. S5A–B and Fig. 2E). This observation suggests that there may be other reasons for the different responses of anti-PD-L1 therapy between young and aged mice.

**Fig. 3:**
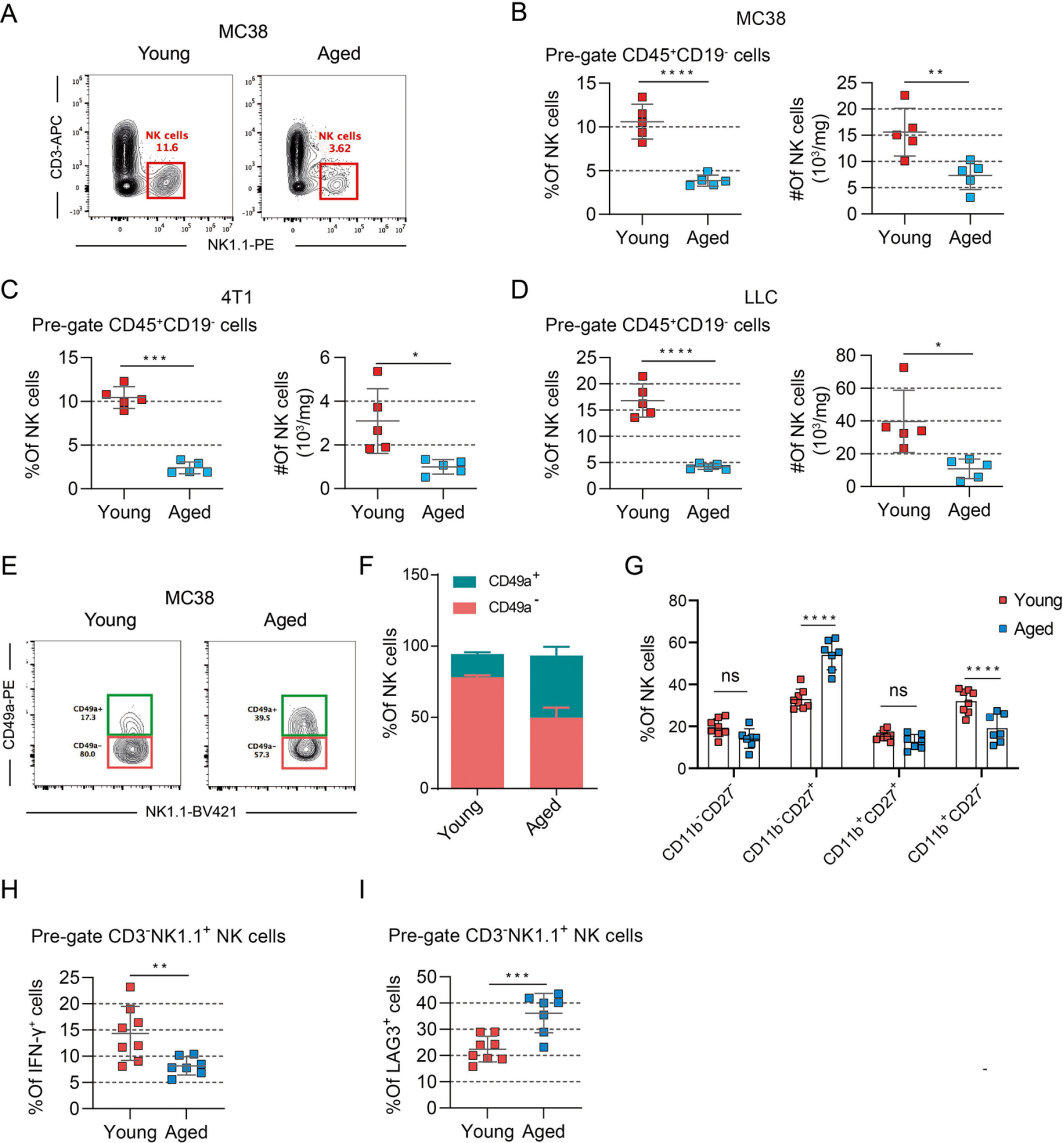
Decreased number and defective function of tumoral NK cells in aged mice. (A) Flow cytometry plots representing of CD3^-^NK1.1^+^ cells proportion among total CD45^+^CD19^−^ cells in MC38 tumors. (B) Quantification of the proportion and absolute number of CD3^−^NK1.1^+^ cells among total CD45^+^CD19^−^ cells in MC38 tumors (*n* = 5 mice per group). (C) Quantification of the proportion and absolute number of CD3^−^NK1.1^+^ cells among total CD45^+^CD19^−^ cells in 4T1 tumors (*n* = 5 mice per group). (D) Quantification of the proportion and absolute number of CD3^−^NK1.1^+^ cells among total CD45^+^CD19^−^ cells in LLC tumors (*n* = 5 mice per group). (E) Flow cytometry plots representing of the frequency of CD49a^+^ cells among total NK cells in MC38 tumors. (F) Quantification of the proportions of CD49a^+^ and CD49^−^ cells among total NK cells from young and aged mice (*n* = 5 mice per group). (G) Quantification of the proportions of CD11b and CD27 expression total NK cells from young and aged mice (*n* = 7–8 mice per group). (H) Quantification of the proportions of IFN-γ expression in total NK cells from young and aged mice (*n* = 7–8 mice per group). (I) Quantification of the proportions of LAG3 expression in total NK cells from young and aged mice (*n* = 7–8 mice per group). Statistical significance was evaluated using independent samples *t*-tests (B-D and H-I) or two-way analysis of variance (ANOVA) (F and G) (*p < 0.05, **p < 0.01, ***p < 0.001, ****p < 0.0001). Error bars represent *SD*

Subsequently, we scrutinized the profile of effector cells associated with antitumor immunity. Compared with that in young mice, the infiltration of NK cells in the tumors of aged mice was significantly lower decreased (Fig. 3A–B), whether in breast cancer (4T1) or lung adenocarcinoma (LLC) (Fig. 3C–D). In addition, differences were observed in NK cell subsets between aged mice and young mice, with a higher prevalence of CD49a^+^ NK cells in aged mice than in young mice (Fig. 3E–F). This CD49a^+^ phenotype is characteristic of tissue-resident tumor NK cells and reflects the interplay between inhibitory molecule expression and immunomodulatory capacity [17, 18]. NK cells can be classified into four populations according to the different expression of CD11b and CD27: from double-negative cells to CD27 single-positive, double-positive cells, and finally to CD11b single-positive, NK cells gradually mature. Previous studies have indicated a significant reduction in NK cell numbers, inadequate maturation of mature NK cells, and compromised NK cell function in aged mice [19]. Then, we examined the maturation of tumor infiltration NK cells in aged mice. We found that the proportion of mature NK cells (CD11b^+^CD27^−^) was decreased, and immature NK cells (CD11b^−^CD27^+^) were increased in aged mice (Fig. 3G). Moreover, the function of IFN-γ by NK cells in aged mice was significantly declined consistent with the increase expression of LAG3^+^ (Fig. 3H–I). There were no differences in NKG2A, CD107a and Ki67 expression between aged mice and young mice (Fig. S6). Our data indicate that the primary disparity in the TME between young and aged mice lies in the diminished presence of NK cells.

### Impaired NK cells lead to insufficient tumor antigen-specific CD8^+^ T cell activation in aged mice

Subsequently, we investigated how NK cells in aged mice influence the efficacy of anti-PD-L1 therapy. Given that NK-derived chemokines are a potent mediator of T cell recruitment [20], we first explored the recruitment ability of CD8^+^ T cells from young and aged mice to tumors. CTV-labeled OT-I cells activated in vitro were injected into MC38-OVA tumor-bearing mice via the tail vein, and CTV^+^ OT-I cell infiltration within the TME was analyzed 48 h post-injection (Fig. S7A). There is no difference in tumor antigen-specific OT-I cells in tumors from aged and young mice (Fig. S7B), suggesting that T cell recruitment may not contribute to resistance to anti-PD-L1 therapy.

**Fig. 4:**
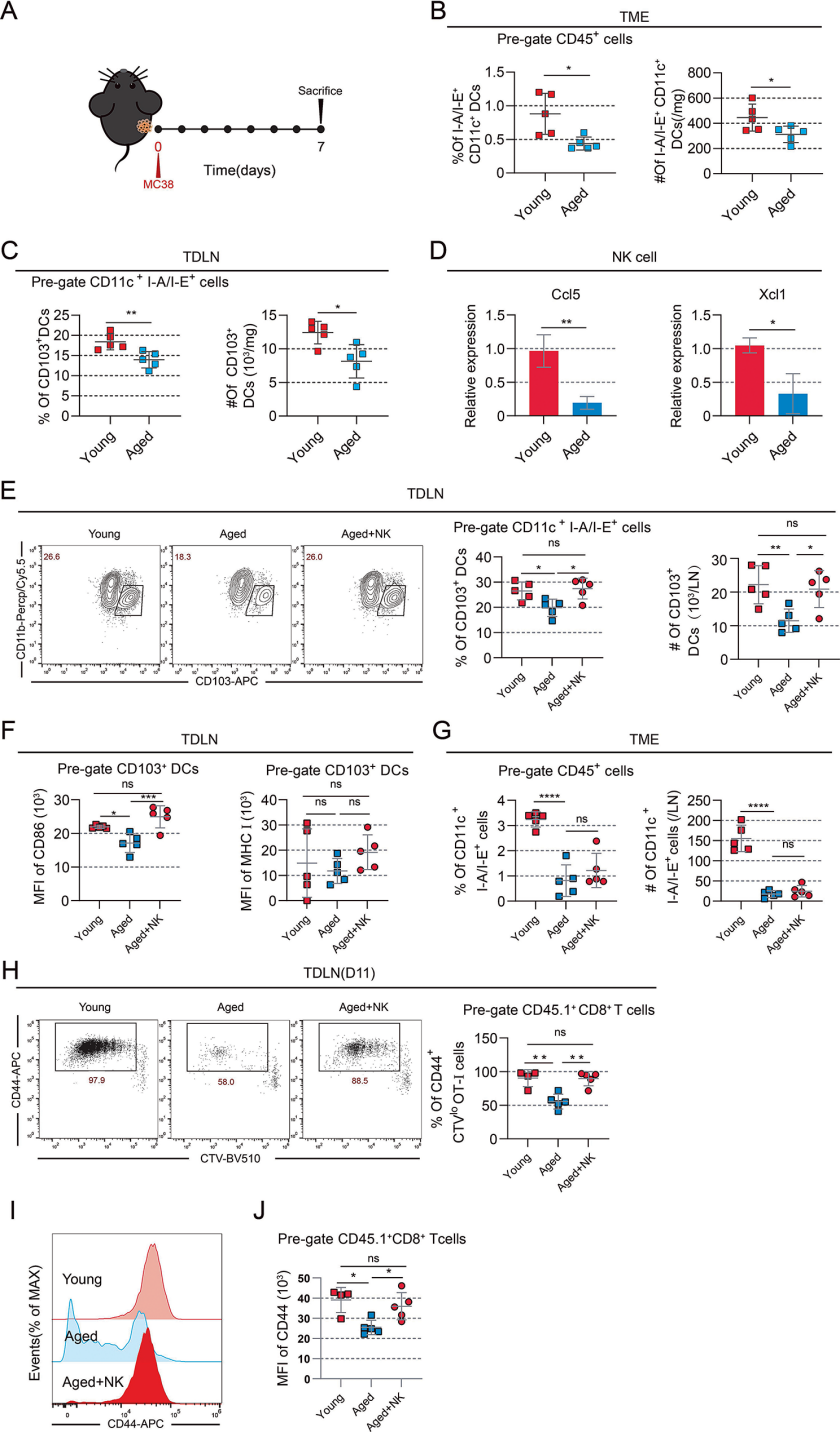
The scarcity of tumoral NK cells contributes to insufficient tumor antigen-specific CD8^+^ T cells priming in aged mice. (A–D) MC38 cells were subcutaneously inoculated into the right flanks of mice, followed by flow cytometry analysis of DCs on day 7. (A) Experimental schedule; (B) the proportion and absolute number of DCs in tumors; (C) the proportion and absolute number of CD103^+^ DCs in TDLNs; (D) relative mRNA expression of Ccl5 and Xcl1 in NK cells in tumors (*n* = 5 mice per group). (E-G) Mice were subjected to subcutaneous injection of 5 × 10^5^ MC38-OVA cells into the right flank, followed by transfer of 1 × 10^6^ NK cells from tumor-free young mice to aged mice on day one post-tumor challenge. The mice were sacrificed on day seven post-tumor challenge, and the right inguinal lymph nodes and tumors were collected for analysis. (E) Representative flow plots and a summary of the proportion and absolute number of CD103^+^ cells in DCs; (F) the mean fluorescence intensity (MFI) of CD86 or MHCI in CD103^+^ DCs; and (G) the proportion and absolute number of DCs within the tumor (*n* = 5 mice per group). (H-J) Mice received subcutaneous injection of 5 × 10^5^ MC38-OVA cells into the right flank, then transferred 1 × 10^6^ NK cells from tumor-free young mice to aged mice on day one post-tumor challenge. On day eight post-tumor challenge, 2 × 10^5^ CTV^+^CD45.1^+^ OT-I cells were injected via the tail. Mice were sacrificed on day 11 of the post-tumor challenge, and the right inguinal lymph nodes were harvested. (H) Representative flow plots and a summary of the proportion of CD44^+^CTV^lo^ OT-I cell; (I) histogram of CD44 in OT-I cells; and (J) the MFI of CD44 in OT-I cells (*n* = 4–5 mice per group). Statistical significance was evaluated using independent samples *t*-tests (B-D) or one-way analysis of variance (ANOVA) (E-H and J) (*p < 0.05, **p < 0.01, ***p < 0.001, ****p < 0.0001). Error bars represent *SD*

As NK cells play a role in facilitating the recruitment of DCs and promoting their survival [14], we hypothesize that the scarcity of NK cells in the tumors of aged mice may lead to the resistance of anti-PD-L1 therapy by affecting DC recruitment. We then analyzed the infiltration of DCs in MC38 tumor models of young and aged mice (Fig. 4A). Both the proportion and quantity of tumor infiltration DCs in aged mice were significantly lower than those in young mice (Fig. 4B). Similarly, the proportion and quantity of CD103^+^ DCs in the TDLN of aged mice were lower than those in young mice (Fig. 4C). Previous studies have demonstrated that NK cell-derived chemokines are essential for DCs recruitment [14, 15], so we examined the chemokines that are important for DCs recruitment in MC38-bearing aged and young mice, such as CCL5 and XCL1. In MC38 tumor tissues, the relative mRNA expression of CCL5 was significantly decreased in aged mice (Fig. S8A), while XCL1 showed a moderate decrease (Fig. S8B). Additionally, the expression of CCL5 and XCL1 in NK cells sorted by flow cytometry also decreases significantly (Fig. 4D).

To elucidate the impact of NK cells on DCs infiltration and naïve T cells activation, we initially transferred NK cells from tumor-free young mice to aged mice. The infiltration of CD103^+^ DCs and its CD86 expression level were both lower in the TDLN of aged mice than those in young mice (Fig. 4E–F). Infusion of NK cells led to an increased infiltration of CD103^+^ DCs and promoted their CD86 but not MHCI expression in the TDLN of aged mice (Fig. 4E–F). However, in tumors, although the infiltration of DCs were significantly reduced in aged mice compared to young mice, infusion of NK cells did not increase the infiltration of DCs in tumors (Fig. 4G). Infusion of CTV-labelled naïve OT-I cells followed by flow cytometry analysis on day 11 revealed a higher proportion of CD44^+^CTV^lo^ OT-I cells in young mice compared to aged mice (Fig. 4H). The expression of CD44 in OT-I cells from aged mice was lower than that of young mice (Fig. 4I–J). Infusing NK cells from young mice could significantly increase the proportion of CD44^+^CTV^lo^ OT-I cells and the expression level of CD44 in OT-I cells, which suggests that NK cell infusion could enhance the initial activation of tumor antigen-specific CD8^+^ T cells in aged mice (Fig. 4H–J). However, following NK cells infusion, there was no change in the relative mRNA expression of CCL5 and XCL1 in the tumor tissues (Fig. S8C–D).

In conclusion, these findings indicate that inadequate NK cell presence in the TME contributes to resistance to anti-PD-L1 therapy in aged mice by impeding the activation of tumor antigen-specific T cells within TDLNs.

### NK cell transfer overcomes resistance to anti-PD-L1 therapy in aged mice by remodeling the TME

**Fig. 5:**
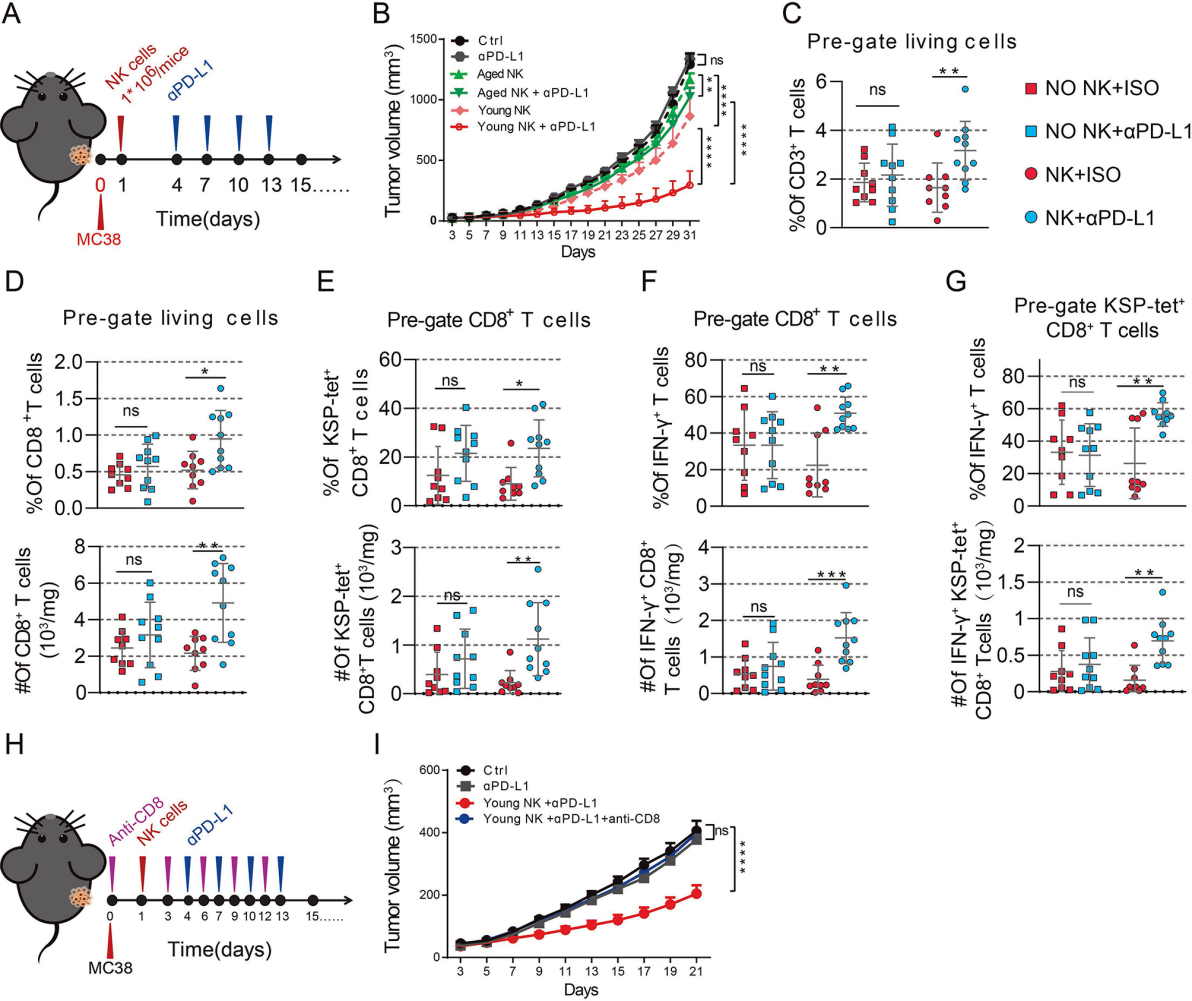
Adoptive transfer of NK cells from young mice overcomes resistance to anti-PD-L1 therapy in aged mice by remodeling the TME. (A and B) Subcutaneous inoculation of MC38 cells into the right flanks of mice. Transfer of NK cells from tumor-free young or aged mice to aged mice on day one post-tumor challenge. Intraperitoneal injection of anti-PD-L1 or control IgG2b on days 4, 7, 10, and 13 after tumor challenge. (A) Experimental schedule; (B) a summary of tumor growth curves(*n* = 6 mice per group). (C-G) Subcutaneous inoculation of MC38 cells into the right flanks of mice. Transfer of NK cells from tumor-free young mice to aged mice on day one post-tumor challenge. Intraperitoneal injection of anti-PD-L1 or control IgG2b on days 4, 7, 10, and 13 after tumor challenge. Mice were sacrificed on day 15 of the post-tumor challenge, and tumors were harvested. (C) Quantification of proportions of CD3^+^ T cells in living cells (*n* = 9–10 mice per group). (D) Quantification of proportion and absolute number of CD8^+^ T cells in living cells (*n* = 9–10 mice per group). (E) Quantification of proportions and absolute number of KSP-tet^+^ CD8^+^ T cells in CD8^+^ cells (*n* = 9–10 mice per group). (F) Quantification of proportions and absolute number of IFN-γ expression in CD8^+^ cells (*n* = 9–10 mice per group). (G) Quantification of proportions and absolute number of IFN-γ expression in KSP-tet^+^ CD8^+^ T cells (*n* = 9–10 mice per group). (H and I) Subcutaneous inoculation of MC38 cells into the right flanks of mice. Transfer of NK cells from tumor-free young mice to aged mice on day one post-tumor challenge. Intraperitoneal injection of anti-CD8a antibodies on days 0, 3, 6, 9, and 12, and anti-PD-L1 or control IgG2b on days 4, 7, 10, and 13 after tumor challenge. (H) Experimental schedule; and (I) a summary of tumor growth curves (*n* = 7 mice per group). Statistical significance was evaluated using two-way analysis of variance (ANOVA) (B and I) or one-way ANOVA (C–G) (^*^p < 0.05, ^**^p < 0.01, ^***^p < 0.001, ^****^p < 0.0001). Error bars represent *SEM* (B, E bottom, G bottom and I) and *SD* (C-G)

To determine the potential of NK cells in anti-PD-L1 therapy in aged mice, we infused NK cells obtained from tumor-free young or aged mice into tumor-bearing aged mice respectively and then administered anti-PD-L1 therapy (Fig. 5A). In accordance with our previous findings, aged mice exhibited resistance to anti-PD-L1 therapy (Fig. 5B). Additionally, adoptive transfer of NK cells from either young or aged mice combined with anti-PD-L1 therapy reduces tumor volumes in aged mice (Fig. 5B), suggesting that NK cell infusion can reverse the therapeutic effect of anti-PD-L1 therapy.

A comprehensive analysis unveiled diverse TME alterations after infusion of young mice-derived NK cells. Notably, there was a significant increase in the infiltration of T cells in aged mice (Fig. 5C), primarily driven by elevated infiltration of CD8^+^ T cells, especially tumor antigen-specific CD8^+^ T cells (Fig. 5D–E). However, the infiltration of CD4^+^ T cells did not show significant changes (Fig. S9C). In addition, the adoptive transfer of NK cells from tumor-free young mice enhanced the cytotoxic cytokine-secreting capacity of total CD8^+^ T cells and tumor antigen-specific CD8^+^ T cells after immunotherapy (Fig. 5F–G), highlighting the role of NK cells from young mice in restoring T cell function. Next, we examined whether NK cell transfer’s effect on anti-PD-L1 therapy efficacy was dependent on CD8^+^ T cells by CD8a neutralizing antibodies. The results showed that ablation of CD8^+^ T cells counteracts the immunotherapy benefit of NK transfer (Fig. 5H–I). Additionally, infusion of NK cells from young mice to aged mice could also decrease the infiltration of immunosuppressive cells, such as TAM, but not MDSCs and Tregs (Fig. S9A-B and D).

Taken together, these results suggested that the adoptive transfer of NK cells from young mice could rejuvenate the TME in aged mice, thereby enhancing the efficacy of anti-PD-L1 therapy.

## Discussion

This work revealed a critical role for physiologic aging in reducing ICB efficacy in murine models of MC38 tumors. In young mice with MC38 tumors, anti-PD-L1 therapy can increase the infiltration and function of tumor-infiltrating CD8^+^ T cells, CD4^+^ Tcon cells, and tumor antigen-specific CD8^+^ T cells, resulting in a robust antitumor response. In aged mice, anti-PD-L1 therapy fails to promote antitumor immune response effectively. Detailed analysis of the TME in young and aged mice revealed that they exhibited similar IFN-γ-associated signaling and immune cell subpopulations, including effector CD8^+^T cells, TAMs, MDSCs, and Tregs. However, the infiltration of NK cells in MC38 tumor-bearing aged mice was reduced by more than half, and a similar phenomenon was observed in 4T1 and LLC tumor models. This resulted in decreased CD103^+^ DCs in TDLNs and a weakened priming of tumor antigen-specific CD8^+^ T cells in aged mice. Excitingly, the adoptive transfer of NK cells from tumor-free young mice can effectively improve the response to immunotherapy.

The incidence of many cancers increases proportionally with age, and cancer become the leading cause of death among individuals aged 60–79 [21]. The emergence of immunotherapy revolutionized the strategies of tumor therapy [22]. However, few elderly patients were recruited for clinical trials of ICB, primarily due to the potential more significant toxicity [23–25]. Furthermore, studies investigating the efficacy of ICB in elderly patients have yielded conflicting results, particular in the study of aNSCLC. For instance, compared to docetaxel chemotherapy, patients < 75 years who received nivolumab treatment exhibited significantly improved OS and PFS. The benefit of nivolumab treatment was reduced in patients ≥ 75 years [26, 27]. However, results from the phase IIIB/IV CheckMate 153 trial offered a contrasting perspective, revealing that older patients had a similar OS to the total patient population [28]. Older patients were associated with longer OS in another trial of atezolizumab treatment, known as the OKA trial [29]. The same phenomenon also occurs in melanoma. In the phase III clinical trial CheckMate 67, the effect of the combination of nivolumab and ipilimumab on the survival of advanced melanoma was investigated. The improvement in OS was found to be smaller in elderly patients [30]. However, in the KEYNOTE-054 clinical trial, elderly patients exhibited similar OS to younger patients [31].However, in the KEYNOTE-006 clinical trial, a more remarkable improvement in OS was observed among elderly patients receiving pembrolizumab treatment [32]. In renal cell carcinoma, it seems that the efficacy of ICB is not affected by age. For instance, CheckMate 025 confirmed the superiority of nivolumab over everolimus in improving survival time, regardless of age [33]. Similar findings were reported in both KEYNOTE-426 and JAVELIN 101 [34, 35]. Meta-analyses encompassing various cancer types also revealed no difference in OS between younger and elderly patients [36, 37].

The different results obtained in the same cancer type can be attributed to factors such as tumor histological type, prior treatment strategies, and PD-L1 expression. Another possible reason is the varying criteria used to define elderly patients across different trials. The clinical studies that found similar or better OS improvement in elderly patients compared to younger patients after receiving ICB tend to define the cutoff age for elderly patients as 65 years old [29–37]. Especially when compared to non-small cell lung cancer (NSCLC), patients diagnosed with melanoma and renal cell carcinoma are generally younger. Elderly patients > 75 years often respond poorly to PD-1 therapy [26, 27]. A meta-analysis exploring the efficacy of anti-PD-(L)1 therapy in cancer patients > 75 years confirmed that elderly patients in this age group do not benefit from PD-(L)1 therapy as much as younger patients [38]. Our clinical data also support this finding, demonstrating that elderly patients with aNSCLC ≥ 75 years are more resistant to anti-PD-1 therapy compared to younger patients under 75 years. These results further emphasize the significant impact of age on ICB outcomes.

Indeed, the immune microenvironment changes significantly as individuals age [8]. Differences in the cellular composition of the immune microenvironment are crucial to cancer progression and the effectiveness of ICB [2–4]. Consequently, in animal models, factors such as mouse strain, age, and tumor cell type can all influence the composition of the immune microenvironment [8], thereby affecting the therapeutic outcome of ICB. In the context of melanoma treatment, a more significant number of Tregs in young mice may account for the favorable response to anti-PD-1 therapy in aged mice [39]. On the contrary, a decrease in Tregs was observed in an aged mouse model of triple-negative breast cancer [40]. However, no difference in Tregs was observed in our MC38 tumor model. In addition, we also discovered that the tumor growth rate in aged mice was slower than young mice. This same outcome was observed in the model as well [40]. This could be related to diminished tumor angiogenesis [41], senescence of tumor-infiltrating fibroblasts [42], attenuated apoptosis during tumor cell proliferation [43], and compromised stem cell potential [44]. This indicates that not only does the immune system undergo changes with age, but there are also systemic changes throughout the body.

Next, we explored whether the age-related differences in ICB observed in the MC38 tumor model were due to NK cells. Although there was a certain increase in the proportion of CD49a^+^ NK cells and immature NK cells, the difference in the absolute numbers of these cell subsets was not as significant as the overall decrease in NK cell numbers in aged mice. Therefore, we primarily focused on the impact of changes in NK cell numbers on immunotherapy. Subsequently, we conducted an NK cell adoptive transfer experiment. The results showed that compared to the control group, adoptive transfer of NK cells from either aged or young mice enhanced the response to anti-PD-L1 therapy in aged mice. These findings demonstrate that the age-related differences in anti-PD-L1 therapy observed in the MC38 tumor model were due to the reduced infiltration of NK cells in tumors of aged mice. Moreover, our findings also revealed a reduction in tumor-infiltrating and TDLN DCs in aged mice, thereby hampering the effective activation of tumor antigen-specific CD8^+^ T cells within the TDLN. NK cells promote DC infiltration into the TME through dual pathways. First, NK cells directly stimulate tumor cell lysis, liberate antigens, and consequently stimulate the migration of DCs. Second, NK cells exert immunomodulatory effects by secreting chemokines such as CCL5, XCL1/2, and FLT3LG to facilitate DC recruitment, thereby augmenting antigen presentation [14, 15, 20]. Our data suggested that aged NK cells express less CCL5 and XCL1, two crucial cytokines involving DC recruitment. Despite the lack of observed changes in chemokines after the infusion of NK cells, which may be because DC-associated chemokines secreted by NK cells in the tumor are localized and diluted in the overall level of the TME, it is essential to note that we observed less migratory CD103^+^ DCs in the TDLN of aged mice. In addition, we found NK cell transfer overcomes aging-driven defects in CD103^+^DCs number and activation of tumor-specific CD8^+^T cells within the TDLN. However, we did not observe any changes in the number of DCs in the TME after NK cells transfer, which may be due to the fact that DCs are very dynamic throughout the tumor immune cycle, and when DCs phagocytose antigens in tumors, they immediately express CCR7 which is converted to the TDLN by CCL10, CCL19, and CCL21, and therefore a change in the number of DCs in the tumor after NK infusion was not observed in the tumor. In addition, the underlying mechanism of aging-driven paucity and impaired function of NK cells remains unknown. Overall, these data demonstrate the first evidence that age can affect the number of NK cells in the MC38 tumor model and may be an essential factor in the response to ICB.

The CAR-NK therapy, which involves genetically modifying NK cells with chimeric antigen receptors (CARs), has demonstrated a remarkable ability to enhance the specificity of NK cell killing significantly. Importantly, this therapy does not induce cytokine release syndrome (CRS) or graft-versus-host disease (GVHD) [45–47]. Currently, CAR-NK is achieving exciting results in clinical trials. Moreover, in patients with melanoma, NK cells play a central role in immunotherapy, as evidenced by tumor infiltration, circulating NK cells, and the effectiveness of ICB [15, 48]. Moreover, the number of peripheral circulating NK cells predicts the efficacy of ICB in patients with NSCLC [49]. These findings underscore the crucial role of NK cells in immunotherapy. Our experimental results also suggest that adoptive transfer of NK cells combined with anti-PD-L1 therapy can enhance antitumor efficacy in aged mice. Based on the preclinical results and data presented in this article, it is feasible to stratify elderly cancer patients based on the number of NK cells in their TME. By administering NK cell adoptive transfer, we can potentiate the therapeutic effects of ICB.

Despite presenting some credible findings, this study has certain limitations. The IO cohort was from one center and included patients with a single tumor type. In the future, patients with multiple tumor types should be enrolled from multiple centers to further evaluate and validate the interplay between aging and response to immunotherapy. Furthermore, as stated in the Top 10 Challenges of Cancer Immunotherapy [50], traditional preclinical models cannot reflect the human immune system well. Our colorectal mouse model may not be a good representation of aNSCLC patients in the clinic, so there is an urgent need to develop more preclinical models. We observed differences in the number of NK cells in tumors of aged mice and changes in subpopulations and functions. Whether these changes will also affect the efficacy of ICB in aged mice requires further exploration and research.

## Conclusion

**Fig. 6:**
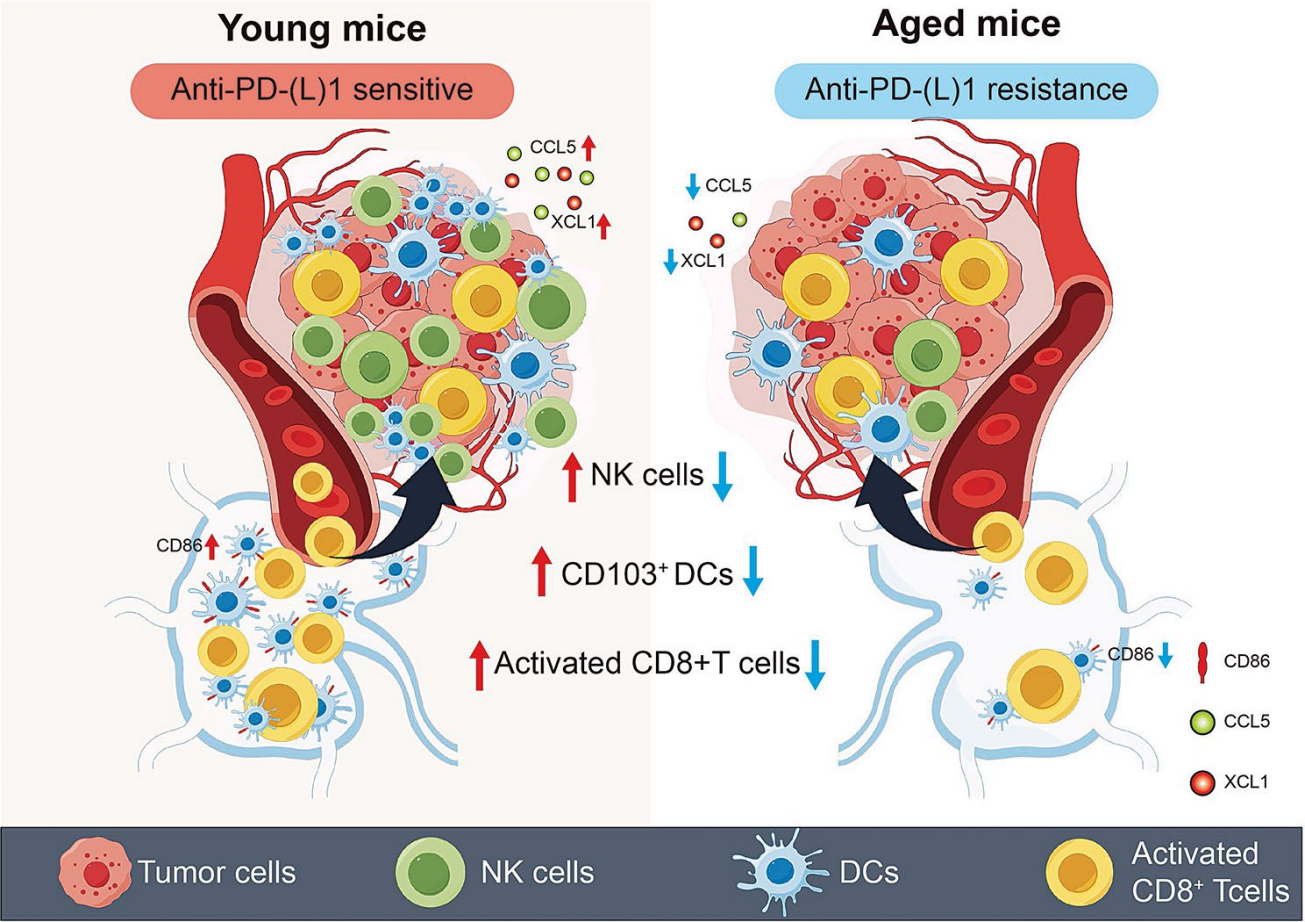
A schematic diagram describes the mechanism of anti-PD-L1 therapeutic resistance in aged tumor-bearing mice. The reduction of NK cells in the tumors of aged mice leads to a decrease in the number of CD103^+^ DCs in the TDLN and a decrease in the expression of CD86 ultimately leading to diminished activation of CD8^+^ T cells

In this work, our data suggest that anti-PD-1 therapy in our IO cohort results in shorter PFS and OS in older patients with aNSCLC. Multiple mouse models have also validated this clinical phenomenon. Resistance to anti-PD-L1 therapy in aged mice is due to decreased NK cells. The reduction of tumoral NK cells in aged mice leads to dampened recruitment and activation of CD103^+^ DC cells in the TDLN, which leads to insufficient CD8^+^ T cell priming and resistance to anti-PD-L1 therapy. Notably, NK cells in young mice can be transferred to remodel the TME to effectively reverse resistance to anti-PD-L1 therapy (Fig. 6). In summary, our study provides a new insight into the mechanism underlying immunotherapy resistance in older patients, paving the way for future research to enhance the response to immunotherapy based on NK cells therapy.

### Electronic supplementary material

Below is the link to the electronic supplementary material.

Supplementary Material 1

## Data Availability

All data are available in the main text or the supplementary materials.

## Declarations

### Author details

^1^Institute of Cancer, Xinqiao Hospital, Third Military Medical University; Chongqing, 400037, China.

^2^Shanghai Pulmonary Hospital, Shanghai, 200082, China.

^3^Department of Oncology, PLA Rocket Force Characteristic Medical Center; Beijing, 100088, China.^4^Jinfeng Laboratory; Chongqing, 401329, China.

## Abbreviations

aNSCLC: Advanced Non-Small Cell Lung Cancer
DCs: Dendritic Cells
ECOG PS: Eastern Cooperative Oncology Group Performance Status
FACS: Fluorescence-Activated Cell Sorting
FasL: Fas Ligand
ICB: Immune Checkpoint Blockade
IFN-γ: Interferon Gamma
MDSCs: Myeloid-Derived Suppressor Cells
MFI: Mean Fluorescence Intensity
NDLN: Non-Draining Lymph Nodes
NK: Natural Killer
NSCLC: Non-Small Cell Lung Cancer
OS: Overall Survival
PD: Progressive Disease
PFS: Progression-Free Survival
PR: Partial Response
SD: Stable Disease
TAMs: Tumor-Associated Macrophages
TDLNs: Tumor-Draining Lymph Nodes
TME: Tumor Microenvironment
TNF-ɑ: Tumor Necrosis Factor alpha
Tregs: Regulatory T cells

## References

[1] Brahmer JR, Tykodi SS, Chow LQ, Hwu WJ, Topalian SL, Hwu P (2012). Safety and activity of anti-PD-L1 antibody in patients with advanced cancer. N Engl J Med.

[2] Gide TN, Quek C, Menzies AM, Tasker AT, Shang P, Holst J (2019). Distinct Immune cell populations define response to Anti-PD-1 monotherapy and Anti-PD-1/Anti-CTLA-4 combined Therapy. Cancer Cell.

[3] Nakamura T, Sato T, Endo R, Sasaki S, Takahashi N, Sato Y, et al. STING agonist loaded lipid nanoparticles overcome anti-PD-1 resistance in melanoma lung metastasis via NK cell activation. J Immunother Cancer. 2021;9(7). 10.1136/jitc-2021-002852. 10.1136/jitc-2021-002852 PMC8256839 34215690

[4] Jia Q, Wang A, Yuan Y, Zhu B, Long H (2022). Heterogeneity of the tumor immune microenvironment and its clinical relevance. Exp Hematol Oncol.

[5] Beerman I, Bhattacharya D, Zandi S, Sigvardsson M, Weissman IL, Bryder D (2010). Functionally distinct hematopoietic stem cells modulate hematopoietic lineage potential during aging by a mechanism of clonal expansion. Proc Natl Acad Sci U S A.

[6] Denkinger MD, Leins H, Schirmbeck R, Florian MC, Geiger H (2015). HSC Aging and Senescent Immune Remodeling. Trends Immunol.

[7] Palmer DB (2013). The effect of age on thymic function. Front Immunol.

[8] Presley CJ, Gomes F, Burd CE, Kanesvaran R, Wong ML (2021). Immunotherapy in older adults with Cancer. J Clin Oncology: Official J Am Soc Clin Oncol.

[9] Raimi-Abraham BT, de Orbe Izquierdo MS, Collignon O, Cerreta F (2017). Regulatory considerations on the enrollment of older adults in oncology clinical trials. J Geriatr Oncol.

[10] Bald T, Krummel MF, Smyth MJ, Barry KC (2020). The NK cell–cancer cycle: advances and new challenges in NK cell–based immunotherapies. Nat Immunol.

[11] Liu S, Galat V, Galat Y, Lee YKA, Wainwright D, Wu J. NK cell-based cancer immunotherapy: from basic biology to clinical development. J Hematol Oncol. 2021;14(1). 10.1186/s13045-020-01014-w. 10.1186/s13045-020-01014-w PMC7788999 33407739

[12] Zhang Y, Zhou W, Yang J, Yang J, Wang W (2023). Chimeric antigen receptor engineered natural killer cells for cancer therapy. Exp Hematol Oncol.

[13] Gong Y, Klein Wolterink RGJ, Wang J, Bos GMJ, Germeraad WTV. Chimeric antigen receptor natural killer (CAR-NK) cell design and engineering for cancer therapy. J Hematol Oncol. 2021;14(1). 10.1186/s13045-021-01083-5. 10.1186/s13045-021-01083-5 PMC8088725 33933160

[14] Bottcher JP, Bonavita E, Chakravarty P, Blees H, Cabeza-Cabrerizo M, Sammicheli S (2018). NK Cells Stimulate Recruitment of cDC1 into the Tumor Microenvironment promoting Cancer Immune Control. Cell.

[15] Barry KC, Hsu J, Broz ML, Cueto FJ, Binnewies M, Combes AJ (2018). A natural killer-dendritic cell axis defines checkpoint therapy-responsive tumor microenvironments. Nat Med.

[16] Ayers M, Lunceford J, Nebozhyn M, Murphy E, Loboda A, Kaufman DR (2017). IFN-gamma-related mRNA profile predicts clinical response to PD-1 blockade. J Clin Invest.

[17] Zhou J, Peng H, Li K, Qu K, Wang B, Wu Y (2019). Liver-Resident NK Cells Control Antiviral Activity of Hepatic T Cells via the PD-1-PD-L1 Axis. Immunity.

[18] Sojka DK, Plougastel-Douglas B, Yang L, Pak-Wittel MA, Artyomov MN, Ivanova Y (2014). Tissue-resident natural killer (NK) cells are cell lineages distinct from thymic and conventional splenic NK cells. Elife.

[19] Shehata HM, Hoebe K, Chougnet CA (2015). The aged nonhematopoietic environment impairs natural killer cell maturation and function. Aging Cell.

[20] Jacobs C, Shah S, Lu WC, Ray H, Wang J, Hockaden N (2024). HSF1 inhibits Antitumor Immune activity in breast Cancer by suppressing CCL5 to Block CD8 + T-cell recruitment. Cancer Res.

[21] Siegel RL, Miller KD, Jemal A (2020). Cancer statistics, 2020. CA Cancer J Clin.

[22] Ma W, Xue R, Zhu Z, Farrukh H, Song W, Li T, et al. Increasing cure rates of solid tumors by immune checkpoint inhibitors. Experimental Hematol Oncol. 2023;12(1). 10.1186/s40164-023-00372-8. 10.1186/s40164-023-00372-8 PMC9843946 36647169

[23] Hutchins LF, Unger JM, Crowley JJ, Coltman CA, Albain KS (1999). Underrepresentation of patients 65 years of age or older in cancer-treatment trials. N Engl J Med.

[24] Townsley CA, Selby R, Siu LL (2005). Systematic review of barriers to the recruitment of older patients with cancer onto clinical trials. J Clin Oncol.

[25] Zhu S, Zhang T, Zheng L, Liu H, Song W, Liu D (2021). Combination strategies to maximize the benefits of cancer immunotherapy. J Hematol Oncol.

[26] Brahmer J, Reckamp KL, Baas P, Crino L, Eberhardt WE, Poddubskaya E (2015). Nivolumab versus Docetaxel in Advanced squamous-cell non-small-cell Lung Cancer. N Engl J Med.

[27] Robert C, Long GV, Brady B, Dutriaux C, Maio M, Mortier L (2015). Nivolumab in previously untreated melanoma without BRAF mutation. N Engl J Med.

[28] Spigel DR, McCleod M, Jotte RM, Einhorn L, Horn L, Waterhouse DM (2019). Safety, Efficacy, and patient-reported Health-Related Quality of Life and Symptom Burden with Nivolumab in patients with Advanced Non-small Cell Lung Cancer, including patients aged 70 years or older or with poor performance status (CheckMate 153). J Thorac Oncol.

[29] Rittmeyer A, Barlesi F, Waterkamp D, Park K, Ciardiello F, von Pawel J (2017). Atezolizumab versus Docetaxel in patients with previously treated non-small-cell lung cancer (OAK): a phase 3, open-label, multicentre randomised controlled trial. Lancet.

[30] Wolchok JD, Chiarion-Sileni V, Gonzalez R, Rutkowski P, Grob JJ, Cowey CL (2017). Overall survival with combined Nivolumab and Ipilimumab in Advanced Melanoma. N Engl J Med.

[31] Eggermont AMM, Blank CU, Mandala M, Long GV, Atkinson V, Dalle S (2018). Adjuvant Pembrolizumab versus Placebo in Resected Stage III Melanoma. N Engl J Med.

[32] Robert C, Schachter J, Long GV, Arance A, Grob JJ, Mortier L (2015). Pembrolizumab versus Ipilimumab in Advanced Melanoma. N Engl J Med.

[33] Motzer RJ, Escudier B, McDermott DF, George S, Hammers HJ, Srinivas S (2015). Nivolumab versus Everolimus in Advanced Renal-Cell Carcinoma. N Engl J Med.

[34] Rini BI, Plimack ER, Stus V, Gafanov R, Hawkins R, Nosov D (2019). Pembrolizumab plus Axitinib versus Sunitinib for Advanced Renal-Cell Carcinoma. N Engl J Med.

[35] Motzer RJ, Penkov K, Haanen J, Rini B, Albiges L, Campbell MT (2019). Avelumab plus Axitinib versus Sunitinib for Advanced Renal-Cell Carcinoma. N Engl J Med.

[36] Nishijima TF, Muss HB, Shachar SS, Moschos SJ (2016). Comparison of efficacy of immune checkpoint inhibitors (ICIs) between younger and older patients: a systematic review and meta-analysis. Cancer Treat Rev.

[37] Elias R, Giobbie-Hurder A, McCleary NJ, Ott P, Hodi FS, Rahma O (2018). Efficacy of PD-1 & PD-L1 inhibitors in older adults: a meta-analysis. J Immunother Cancer.

[38] Nie R-C, Chen G-M, Wang Y, Zhou J, Duan J-L, Zhou Z-W (2021). Efficacy of Anti-PD-1/PD-L1 monotherapy or combinational therapy in patients aged 75 years or older: a study-level Meta-analysis. Front Oncol.

[39] Kugel CH, Douglass SM, Webster MR, Kaur A, Liu Q, Yin X (2018). Age correlates with response to Anti-PD1, reflecting age-related differences in Intratumoral Effector and Regulatory T-Cell populations. Clin Cancer Res.

[40] Sceneay J, Goreczny GJ, Wilson K, Morrow S, DeCristo MJ, Ubellacker JM (2019). Interferon Signaling is diminished with age and is Associated with Immune Checkpoint Blockade Efficacy in Triple-negative breast Cancer. Cancer Discov.

[41] Kaptzan T, Skutelsky E, Itzhaki O, Sinai J, Huszar M, Siegal A (2006). Efficacy of anti-angiogenic treatment of tumors in old versus young mice. Mech Ageing Dev.

[42] Fane M, Weeraratna AT (2020). How the ageing microenvironment influences tumour progression. Nat Rev Cancer.

[43] Leibovici J, Itzhaki O, Kaptzan T, Skutelsky E, Sinai J, Michowitz M (2009). Designing ageing conditions in tumour microenvironment-a new possible modality for cancer treatment. Mech Ageing Dev.

[44] Marsh T, Wong I, Sceneay J, Barakat A, Qin Y, Sjodin A (2016). Hematopoietic age at Onset of Triple-negative breast Cancer dictates Disease aggressiveness and progression. Cancer Res.

[45] Wang X, Yang X, Yuan X, Wang W, Wang Y. Chimeric antigen receptor-engineered NK cells: new weapons of cancer immunotherapy with great potential. Experimental Hematol Oncol. 2022;11(1). 10.1186/s40164-022-00341-7. 10.1186/s40164-022-00341-7 PMC9628181 36324149

[46] Zhang L, Meng Y, Feng X, Han Z (2022). CAR-NK cells for cancer immunotherapy: from bench to bedside. Biomark Res.

[47] Lamers-Kok N, Panella D, Georgoudaki A-M, Liu H, Özkazanc D, Kučerová L, et al. Natural killer cells in clinical development as non-engineered, engineered, and combination therapies. J Hematol Oncol. 2022;15(1). 10.1186/s13045-022-01382-5. 10.1186/s13045-022-01382-5 PMC9644572 36348457

[48] Subrahmanyam PB, Dong Z, Gusenleitner D, Giobbie-Hurder A, Severgnini M, Zhou J (2018). Distinct predictive biomarker candidates for response to anti-CTLA-4 and anti-PD-1 immunotherapy in melanoma patients. J Immunother Cancer.

[49] Mazzaschi G, Facchinetti F, Missale G, Canetti D, Madeddu D, Zecca A (2019). The circulating pool of functionally competent NK and CD8 + cells predicts the outcome of anti-PD1 treatment in advanced NSCLC. Lung Cancer.

[50] Hegde PS, Chen DS (2020). Top 10 challenges in Cancer Immunotherapy. Immunity.

