# Supplementary material for: NK cell transfer overcomes resistance to PD-(L)1 therapy in aged mice
Source: Exp Hematol Oncol. 2024 May 9;13:48. doi: 10.1186/s40164-024-00511-9 (PMC11080179; doi:10.1186/s40164-024-00511-9)
Supplement: Supplementary file 1 — Supplementary Material 1 [file 40164_2024_511_MOESM1_ESM.docx]

Supplementary Materials for

**NK cell transfer overcomes resistance to PD-(L)1 therapy in aged mice**

Junlei Hou^1, #^, Shuanglong Xie^1, 4, #^, Jianbao Gao^1, #^, Tao Jiang^2^, Enjian Zhu^1^, Xuezhi Yang^1^, Zheng Jin^1^, Haixia Long^1^, Anmei Zhang^1^, Fei Yang^1^, Lujing Wang^1^, Haoran Zha^3^, Qingzhu Jia^1, *^, Bo Zhu^1, *^, Xinxin Wang^1, *^

Correspondence to: [wangx82@tmmu.edu.cn](mailto:wangx82@tmmu.edu.cn), [bo.zhu@tmmu.edu.cn](mailto:bo.zhu@tmmu.edu.cn), [qingzhu.jia@tmmu.edu.cn](mailto:qingzhu.jia@tmmu.edu.cn)

**This PDF file includes:**

Supplementary Fig. S1 to S9

Supplementary Tables. S1 to S2

**Supplementary Figures**


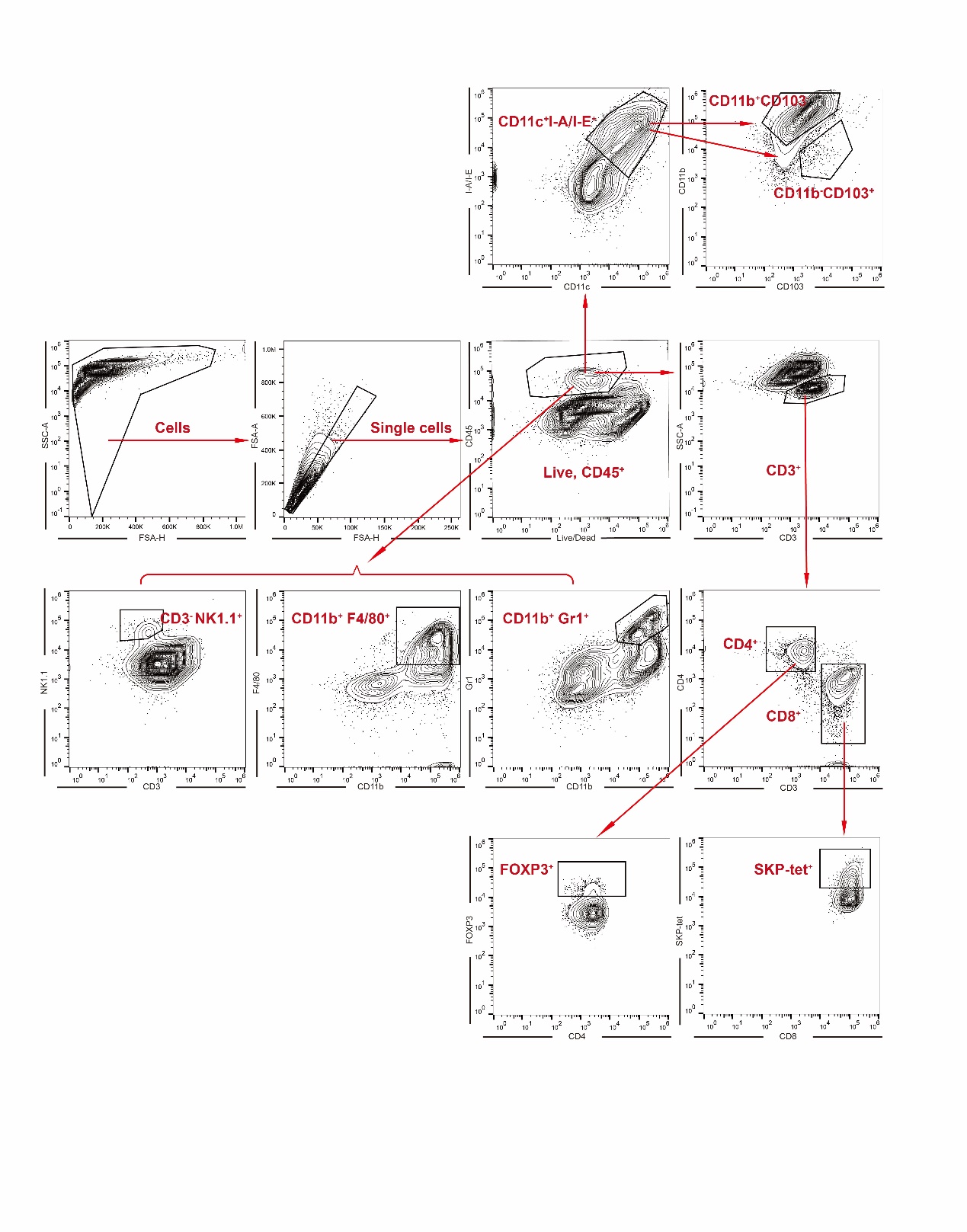
 **Fig. S1 Fluorescence-activated cell sorting (FACS) gating strategy for tumor-infiltrating immune cells.**


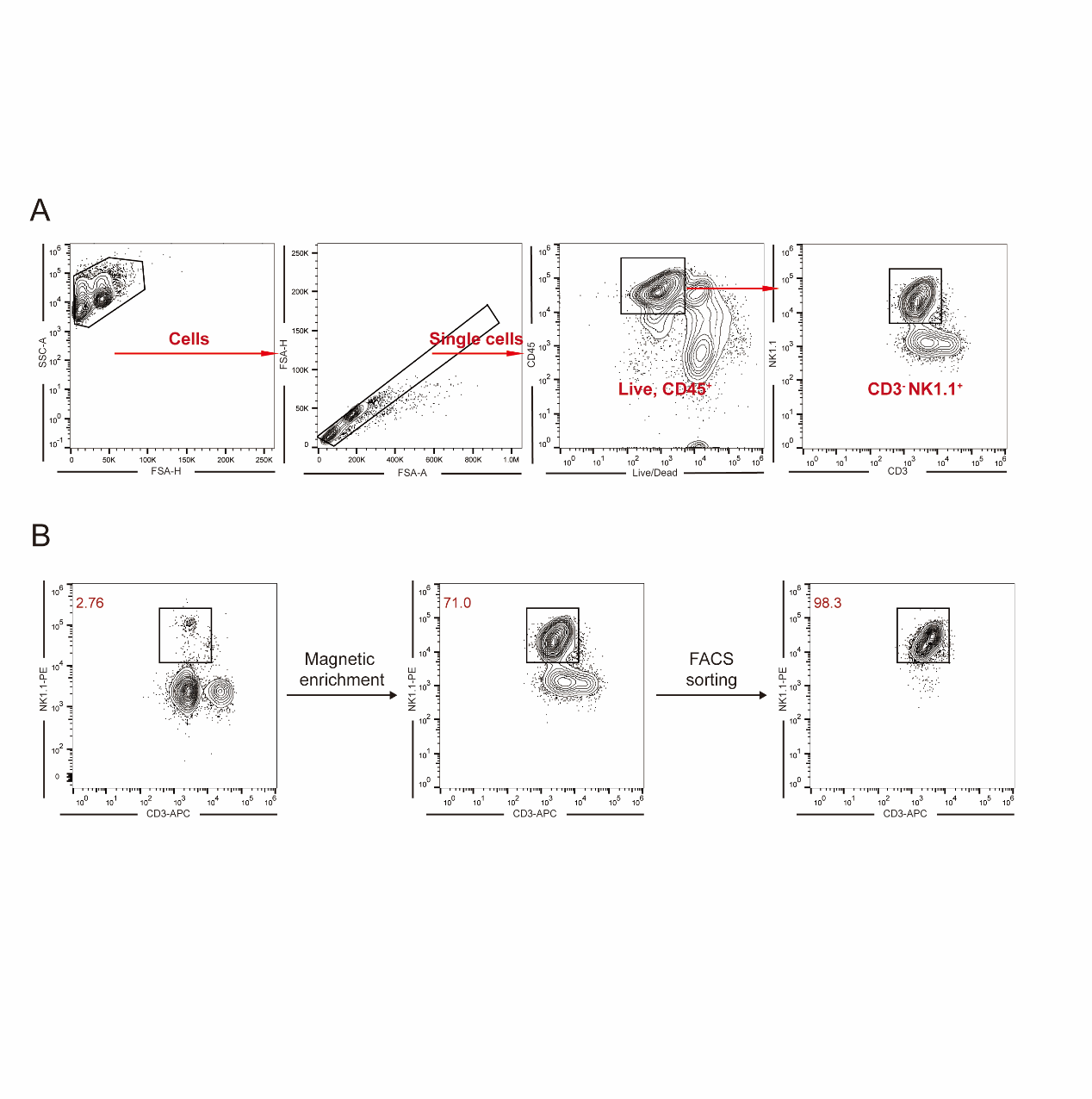


**Fig. S2** **The purity of the transfer NK cells.**

1. FACS gating strategy for NK cells.

(B) The representative flow plot of NK cells purity.


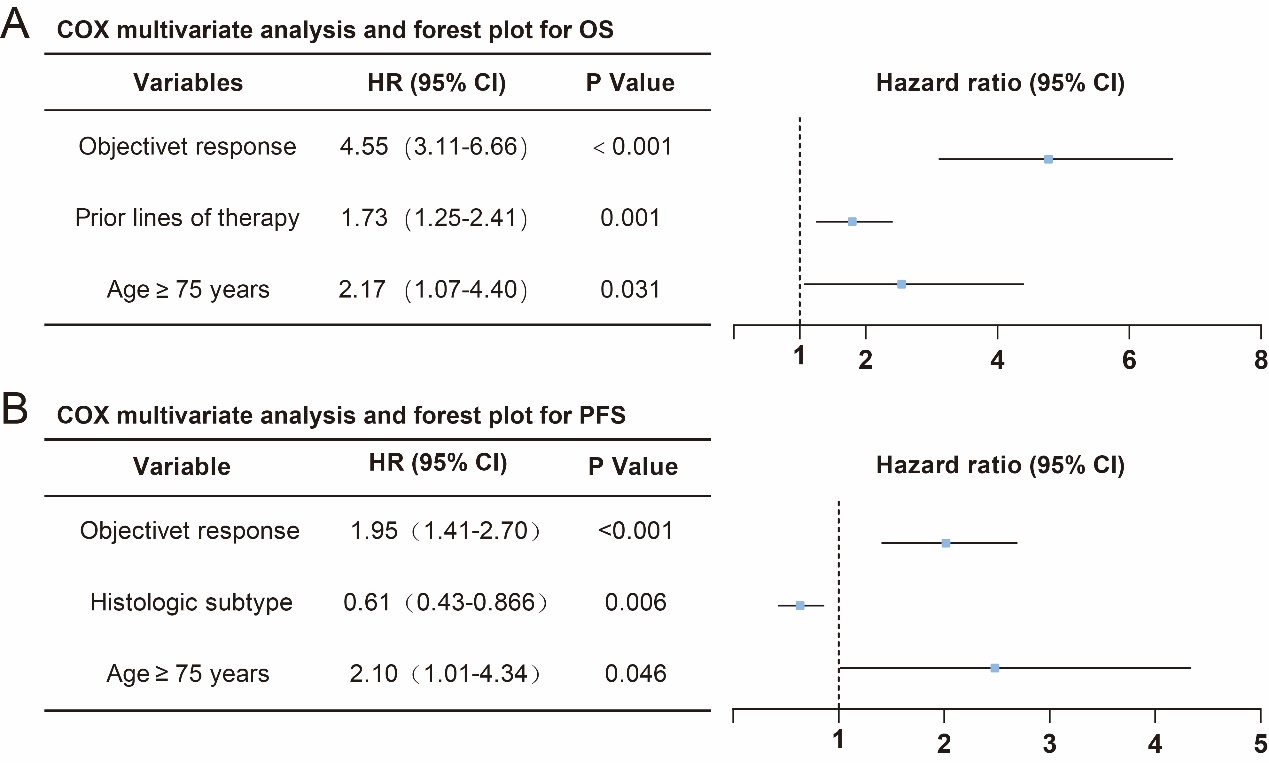


**Fig. S3** **(related to Fig. 1) Multivariable Cox regression analysis and forest plot of factors associated with survival in patients with advanced non-small cell lung cancer.**

(A) Cox multivariate analysis and forest plot for OS.

(B) Cox multivariate analysis and forest plot for PFS.


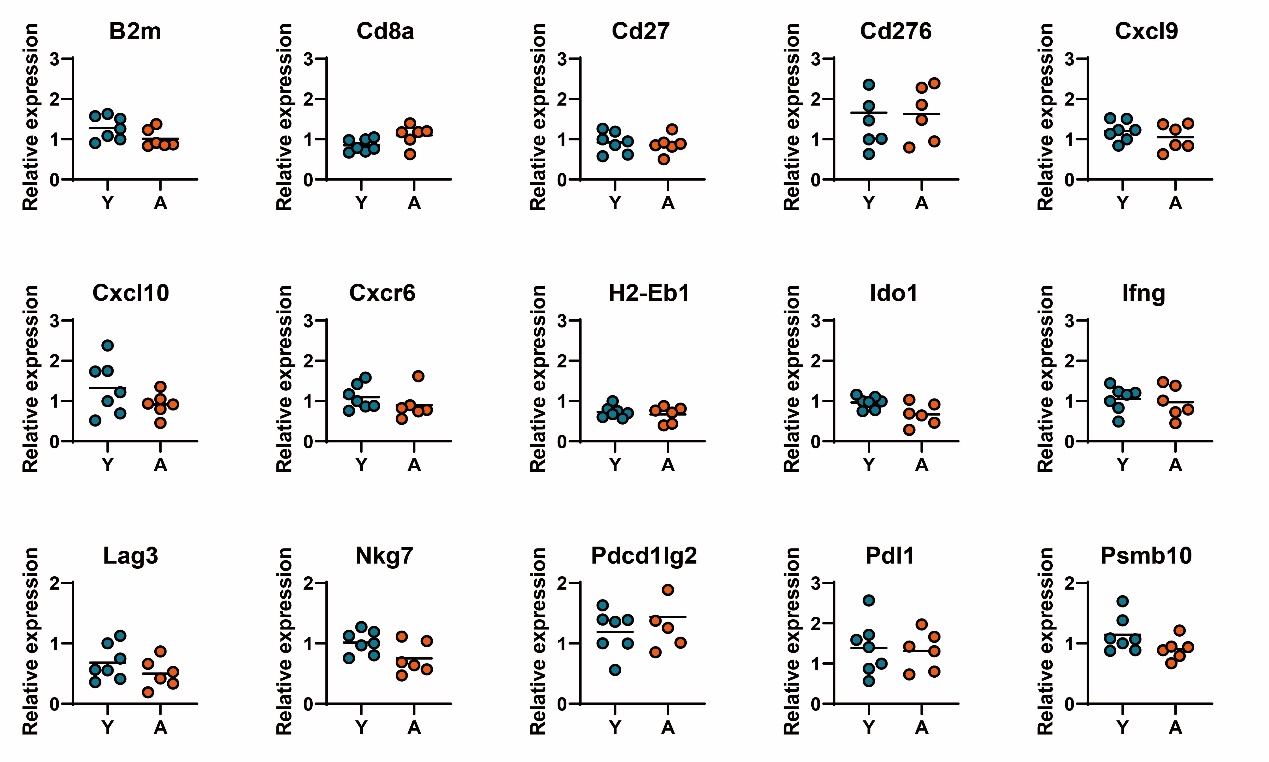


**Fig. S4** **(related to Fig. 3) The differential expression of interferon gamma (IFN-γ)-related genes between young and aged mice.**

Independent sample *t*-tests were performed to evaluate statistical significance (n=6-7 mice per group). None of the differences between young and aged mice were statistically significant (p > 0.05).


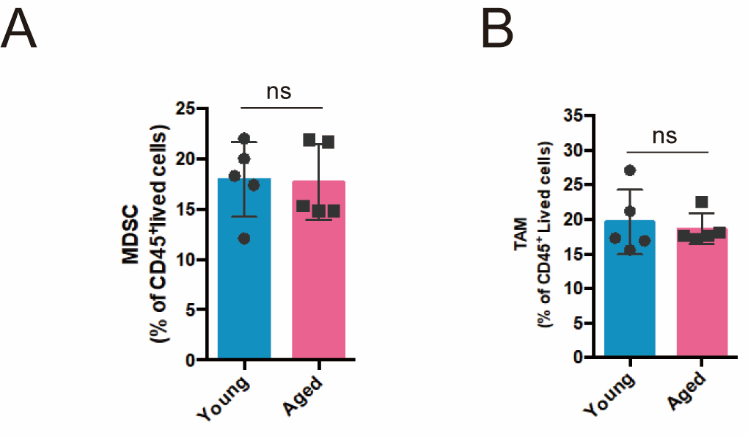


**Fig. S5 (related to Fig. 3) The difference of immune cell infiltration between young and aged mice.**

(A) Quantification of CD11b^+^ Gr1^+^ cells proportions in CD45^+^ lived cells (n=5 mice per group).

(B) Quantification of CD11b^+^ F4/80^+^ cells proportions in CD45^+^ lived cells (n=5 mice per group).

Statistical significance was evaluated using independent samples *t*-tests (^*^p<0.05, ^**^p<0.01, ^***^p<0.001, ^****^p<0.0001). Error bars represent *SD*.


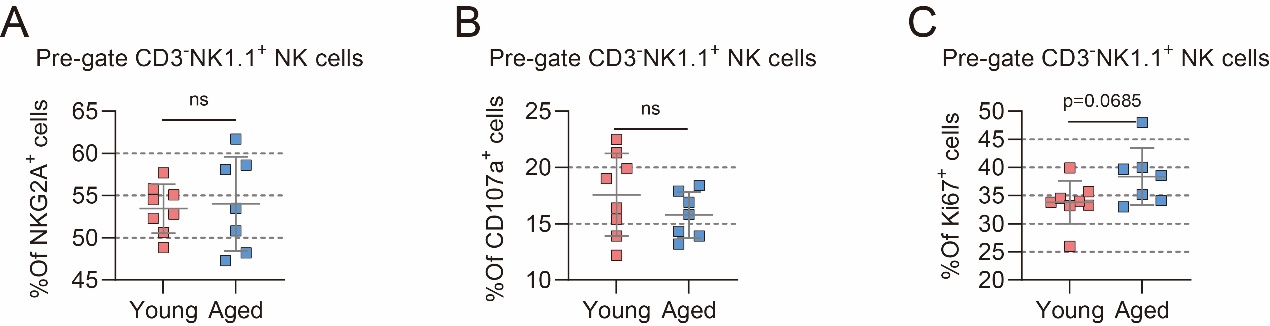


**Fig. S6** **(related to Fig. 3) Phenotypes of NK cells in aged and young mice.**

(A) Quantification of NKG2A expression proportions in the total NK cells from young and aged mice (n=7-8 mice per group).

(B) Quantification of CD107a expression proportions in the total NK cells from young and aged mice (n=7-8 mice per group).

(C) Quantification of Ki67 expression proportions in the total NK cells from young and aged mice (n=7-8 mice per group).

Statistical significance was all evaluated using independent samples *t*-tests (^*^p<0.05, ^**^p<0.01, ^***^p<0.001, ^****^p<0.0001). Error bars represent *SD*.


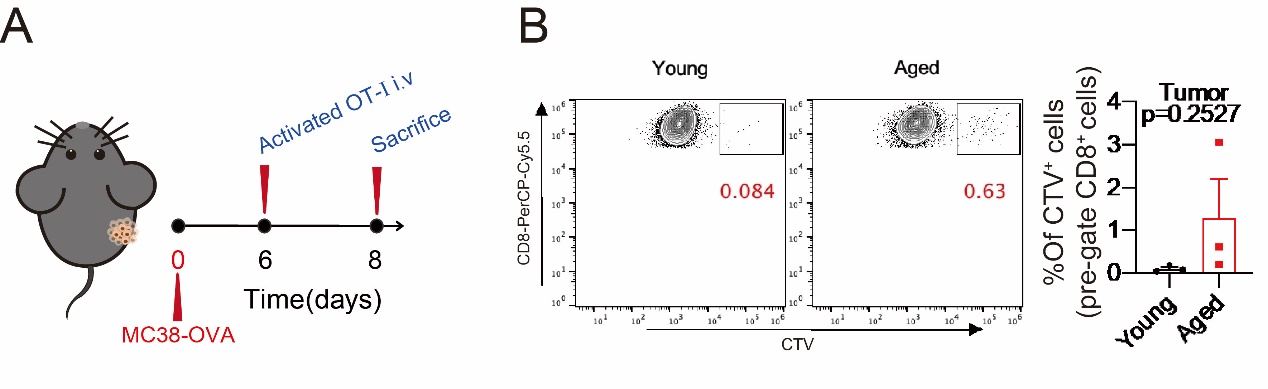


**Fig. S7 (related to Fig. 4) Activated tumor antigen-specific CD8^+^ T cells from aged mice were effectively recruited into the TME.**

(A and B) Subcutaneous inoculation of MC38-OVA cells into the right flank of mice. Active CTV^+^OT-1 cells injected via tail on day six post-tumor challenge. Flow cytometry analysis of tumor-infiltrating OT-1 cells on day eight. (A) Experimental schedule, (B) flow plots and the percentages of CTV^+^ cells in CD8^+^ T cells (n=3 mice per group).

Statistical significance was evaluated using independent samples *t*-tests (^*^p<0.05, ^**^p<0.01, ^***^p<0.001, ^****^p<0.0001). Error bars represent *SD*.


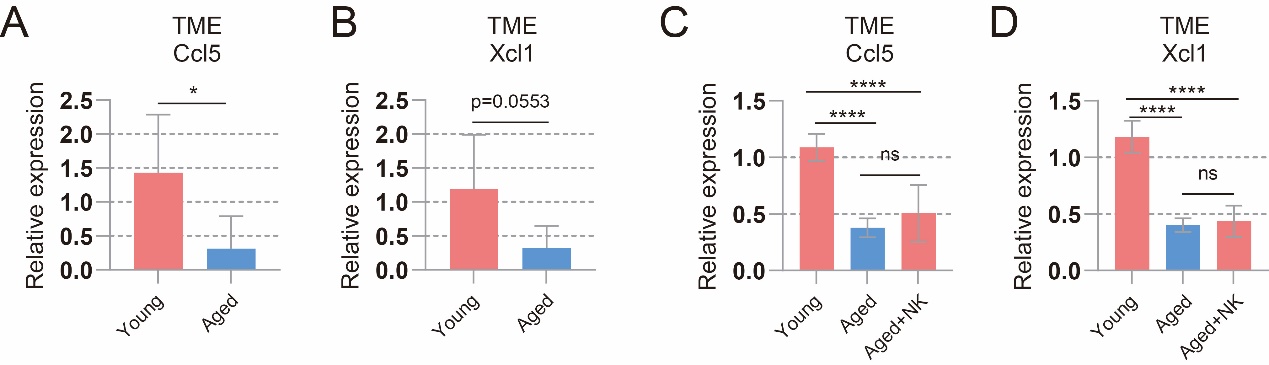


**Fig. S8** **(related to Fig. 4) DC-associated chemokines in the tumor tissues.**

(A) Relative mRNA expression of Ccl5 in tumor tissues (n=3 mice per group).

(B) Relative mRNA expression of Xcl1 in tumor tissues (n=3 mice per group).

(C) Relative mRNA expression of Ccl5 in tumor tissues (n=6-8 mice per group).

(D) Relative mRNA expression of Xcl1 in tumor tissues (n=6-8 mice per group).

Statistical significance was evaluated using independent samples t-tests (A & B) or one-way analysis of variance (ANOVA) (C & D) (*p<0.05, **p<0.01, ***p<0.001, ****p<0.0001). Error bars represent *SD*.


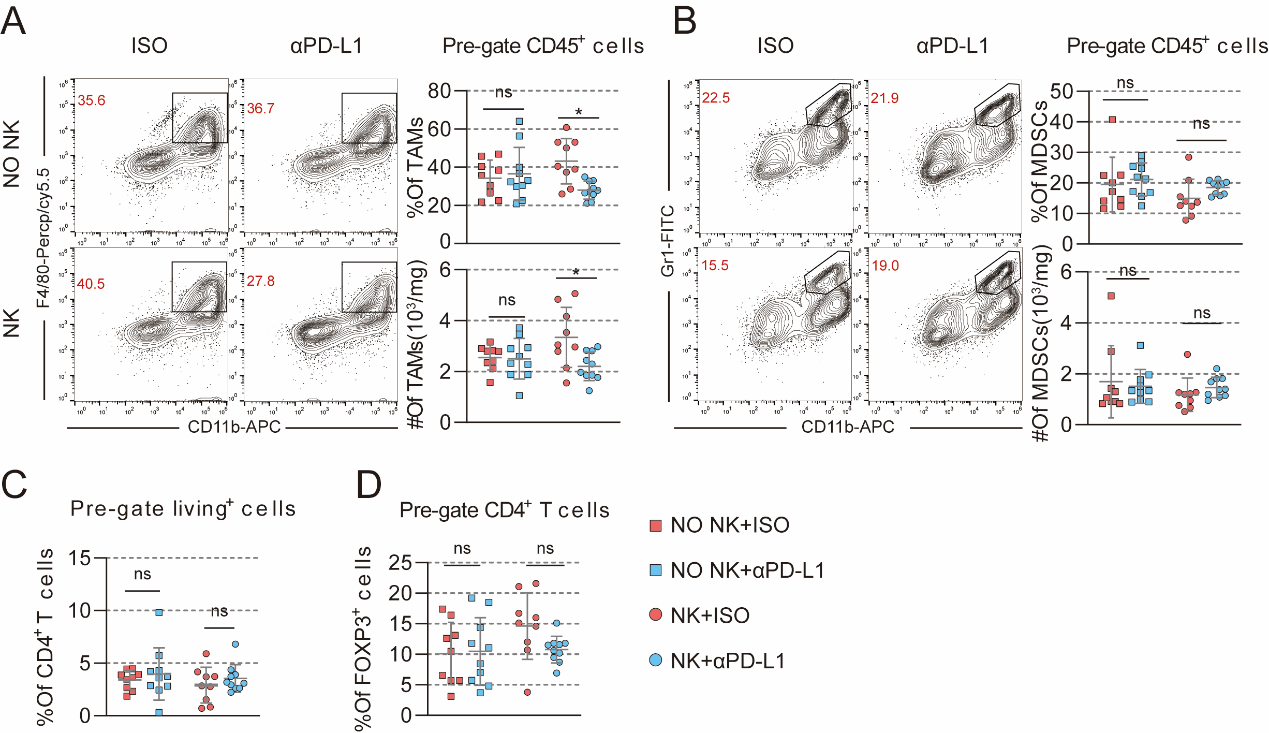


**Fig. S9 (related to Fig. 5) The difference of immune cell infiltration in different experimental groups.**

The mice were treated as described in Fig. 5. Tumors were removed on day fifteen post tumor inoculation and analyzed for tumor-infiltrating cells using flow cytometry.

(A) Flow plots and a summary of the proportion and absolute number of CD11b^+^ F4/80^+^ cells in CD45^+^ cells are shown (n=9–10 mice per group).

(B) Flow plots and a summary of the proportion the absolute number of CD11b^+^ Gr1^+^ cells in CD45^+^ cells are shown (n=9–10 mice per group).

(C) Quantification of the CD4^+^ cells proportion in living cells (n=9–10 mice per group).

(D) Quantification of the FOXP3 proportion in CD4^+^ cells (n=9–10 mice per group).

Statistical significance was evaluated using one-way analysis of variance (ANOVA) (^*^p<0.05, ^**^p<0.01, ^***^p<0.001, ^****^p<0.0001). Error bars represent *SD*.

**Supplementary Tables**

**Table S1. Baseline patient and disease characteristics**

| **Characteristic** | **All Patients**  **(N=205)** | **<75 years**  **(n=191)** | **≥75 years**  **(n=14)** |
| --- | --- | --- | --- |
| **Age, years** | | | |
| Median (range) | 63 (20-89) | 62 (20-73) | 79 (75-89) |
| <75, n (%) | 191 (93) | 191 (100) | 0 (0) |
| ≥75, n (%) | 14 (7) | 0 (0) | 14 (100) |
| **Male, n (%)** | 170 (83) | 157 (82) | 13(93) |
| **Immune Optimal Efficacy, n (%)** | | | |
| PR | 53 (26) | 51 (27) | 2 (14) |
| SD | 90 (44) | 83 (43) | 7 (50) |
| PD | 62 (30) | 57 (30) | 5 (36) |
| **ECOG PS, n (%)** | | | |
| 0 | 17 (8) | 16 (9) | 1 (7) |
| 1 | 178 (87) | 167 (87) | 11 (79) |
| 2 | 10 (5) | 8 (4) | 2 (14) |
| **Smoking status, n (%)** | | | |
| Never | 74 (36) | 71 (37) | 3 (21) |
| Current | 65 (32) | 60 (31) | 5 (36) |
| Former | 63 (31) | 57 (30) | 6 (43) |
| Not reported | 3 (1) | 3 (2) | 0 (0) |
| **Disease stage, n (%)** | | | |
| Ⅲ | 28 (14) | 25 (13) | 3 (21) |
| Ⅳ | 177 (86) | 166 (87) | 11 (79) |
| **Histologic subtype, n (%)** | | | |
| Squamous | 64 (31) | 56 (29) | 8 (57) |
| Adenocarcinoma | 121 (59) | 116 (61) | 5 (36) |
| Other | 20 (10) | 19 (10) | 1 (7) |
| **Prior line of therapy, n (%)** | | | |
| 1 | 59 (29) | 56 (29) | 3 (21) |
| 2 | 95 (46) | 89 (47) | 6 (43) |
| ≥3 | 49 (24) | 44 (23) | 5 (36) |
| Not reported | 2 (1) | 2 (1) | 0 (0) |

ECOG PS, Eastern Cooperative Oncology Group performance status; PD, progressive disease; PR, partial response; SD, stable disease

**Table S2. List of primers used in this study**

| Gene name | Forward (5′-3′) | Reverse (5′-3′) |
| --- | --- | --- |
| B2m | TTCTGGTGCTTGTCTCACTGA | CAGTATGTTCGGCTTCCCATTC |
| Cd8a | CCGTTGACCCGCTTTCTGT | CGGCGTCCATTTTCTTTGGAA |
| Cd27 | AGAAGAAACCACGGGCCAAAT | CTCCTGGATAGGGATAGCACTG |
| Cd276 | ATGCTTCGAGGATGGGGTG | CCAGGCTCTGGGGAAAAGG |
| Cxcl9 | TCCTTTTGGGCATCATCTTCC | TTTGTAGTGGATCGTGCCTCG |
| Cxcl10 | CCAAGTGCTGCCGTCATTTTC | GGCTCGCAGGGATGATTTCAA |
| Cxcr6 | GAGTCAGCTCTGTACGATGGG | TCCTTGAACTTTAGGAAGCGTTT |
| H2-Eb1 | GCGGAGAGTTGAGCCTACG | CCAGGAGGTTGTGGTGTTCC |
| Ido1 | GCTTTGCTCTACCACATCCAC | CAGGCGCTGTAACCTGTGT |
| Ifng | ATGAACGCTACACACTGCATC | CCATCCTTTTGCCAGTTCCTC |
| Lag3 | CTGGGACTGCTTTGGGAAG | GGTTGATGTTGCCAGATAACCC |
| Nkg7 | TCAAGTCCAGACATTCTTCTCCT | CACAAGGTTTCATACTCAGCCC |
| Pdcd1lg2 | CTGCCGATACTGAACCTGAGC | GCGGTCAAAATCGCACTCC |
| Pdl1 | GCTCCAAAGGACTTGTACGTG | TGATCTGAAGGGCAGCATTTC |
| Psmb10 | GAGGAATGCGTCCTTGGAACA | CACAACCGAATCGTTAGTGGC |
| Ccl5 | GCTGCTTTGCCTACCTCTCC | TCGAGTGACAAACACGACTGC |
| Xcl1 | TTTGTCACCAAACGAGGACTAAA | CCAGTCAGGGTTATCGCTGTG |
| β-actin | CCTGAGGCTCTTTTCCAGCC | AGAGGTCTTTACGGATGTCAACGT |
